# First-line treatment for advanced or metastatic EGFR mutation-positive non-squamous non-small cell lung cancer: a network meta-analysis

**DOI:** 10.3389/fonc.2024.1498518

**Published:** 2025-01-15

**Authors:** Mengyao Zhang, Lan Sun

**Affiliations:** Department of Oncology, Bishan Hospital of Chongqing Medical University, Chongqing, China

**Keywords:** EGFR mutation-positive, non-small cell lung cancer, non-squamous, first-line treatment, network meta-analysis

## Abstract

**Background:**

Several head-to-head meta-analyses have compared the efficacy and safety of different first-line treatments in patients with EGFR mutation-positive (M+) advanced or metastatic non-squamous non-small cell lung cancer (nsq-NSCLC). However, there is a lack of comprehensive evaluation encompassing multiple treatment strategies. Our objective is to conduct a network meta-analysis that includes various treatment modalities, enabling both direct and indirect comparisons for a more thorough assessment.

**Methods:**

We conducted a search of PubMed, Embase, Cochrane Library, and Web of Science databases from inception until May 8, 2024, to identify eligible randomized controlled trials (RCTs). The primary endpoints were progression-free survival (PFS) and overall survival (OS), while secondary outcomes included objective response rate (ORR) and grade 3 or higher adverse events (≥3AEs). Stata 15.0 and R 4.3.2 software were utilized for the network meta-analysis.

**Results:**

A total of 30 RCTs, comprising 8654 participants, were included. The study encompassed the following 19 treatments: Chemotherapy; Afatinib; Afatinib + Cetuximab; Apatinib + Gefitinib; Befotertinib; Cetuximab + Chemotherapy; Erlotinib; Erlotinib + Bevacizumab; Erlotinib + Chemotherapy; Gefitinib; Gefitinib + Chemotherapy; Gefitinib + Olaparib; Icotinib; Icotinib + Chemotherapy; Lazertinib; Naquotinib; Osimertinib; Osimertinib + Bevacizumab; Osimertinib + Chemotherapy. The network meta-analysis results indicated that, in terms of PFS, Osimertinib + Chemotherapy (SUCRAs: 93.4%) and Osimertinib (SUCRAs: 84.61%) were the most effective. Regarding OS, Lazertinib (SUCRAs: 89.72%), Gefitinib (SUCRAs: 72.07%), and Osimertinib + Chemotherapy (SUCRAs: 70.74%) emerged as the top three options. Afatinib (SUCRAs: 92.27%) was associated with the best ORR improvement. For ≥3AEs, Afatinib (SUCRAs: 74.93%) and Osimertinib (SUCRAs: 69.42%) were likely the best choices.

**Conclusion:**

Current evidence suggests that, considering both survival and safety, Osimertinib stands out as the preferred first-line treatment for untreated EGFR M + advanced or metastatic nsq-NSCLC. Notably, the combination of Osimertinib with chemotherapy demonstrated superior survival benefits. However, due to the limitations in the number and quality of included studies, these conclusions await further validation through more high-quality research.

**Systematic review registration:**

https://www.crd.york.ac.uk/prospero/display_record.php?ID=CRD42024562981, identifier CRD42024562981.

## 1 Introduction

Lung cancer is a global health issue, with non-small cell lung cancer (NSCLC) being the most common type, accounting for approximately 80%-85% of cases. Most patients are diagnosed at an advanced stage (1, 2). About 70% of NSCLC cases are diagnosed at a late stage, characterized by poor prognosis, with a 5-year survival rate of only 26% (3). NSCLC can be further subdivided into two main histological subtypes: squamous NSCLC and non-squamous NSCLC, with the latter being the major subtype, accounting for 70%-75% of all NSCLC cases (4). In the progression of NSCLC, activating mutations in the epidermal growth factor receptor (EGFR) play a central role, particularly exon 19 deletions (ex19del) and exon 21 L858R mutations. Globally, the prevalence of EGFR mutations is approximately 32%, with a significantly higher prevalence in Asians (40%-60%) compared to Western NSCLC populations (10%-15%). Additionally, the mutation rate is higher in non-squamous NSCLC patients compared to squamous NSCLC patients (5, 6). The discovery of EGFR mutations has ushered in the era of targeted therapy for lung cancer, making tumor cells more sensitive to tyrosine kinase inhibitors (TKIs).

In recent years, first-line treatment options for patients with EGFR mutation-positive non-squamous NSCLC have undergone significant advancements. First-generation EGFR-TKIs (such as gefitinib, erlotinib, and icotinib) and second-generation EGFR-TKIs (such as afatinib and dacomitinib) have established their superiority over platinum-based chemotherapy in the clinical practice for untreated advanced EGFR-mutant NSCLC patients (7–10). However, most patients develop acquired resistance to these treatments within approximately one year, with the most common mechanism being the EGFR T790M mutation (11). To overcome T790M-mediated resistance, third-generation EGFR-TKIs, such as osimertinib and lorlatinib, have been developed. Osimertinib is effective not only against common EGFR-sensitive mutations but also selectively inhibits the T790M resistance mutation, demonstrating significant clinical efficacy and manageable safety (12–14). Furthermore, combination therapies are being explored in addition to monotherapy. For example, combinations of EGFR-TKIs with anti-angiogenic agents such as bevacizumab, immune checkpoint inhibitors, or platinum-based chemotherapy are being investigated to further improve objective response rates (ORR) and prolong progression-free survival (PFS) (15). However, there is ongoing debate on whether combination therapy should be established as the standard first-line treatment.

Despite a few head-to-head meta-analyses comparing the efficacy and safety of different first-line treatment options in patients with EGFR-mutant advanced non-squamous NSCLC, these analyses focus on pairwise comparisons and lack a comprehensive consideration of multiple treatment strategies (16, 17). Our aim is to conduct a network meta-analysis (NMA) that simultaneously includes multiple treatment modalities for direct and indirect comparisons, providing a more comprehensive efficacy evaluation to better support clinical decision-making.

## 2 Methods

We followed the Preferred Reporting Items for Systematic Reviews and Meta-Analyses (PRISMA) guidelines for reporting systematic reviews and meta-analyses, and their requirements for NMA (18). The study protocol was registered on the International Prospective Register of Systematic Reviews (PROSPERO) (CRD42024562981).

### 2.1 Search strategy

The search strategy combined subject terms and free terms, searching four electronic databases: PubMed, Web of Science, Cochrane Library, and Embase. The search covered the period from the establishment of the databases until May 8, 2024, with the language limited to English. Additionally, to minimize the risk of omission, we cross-referenced review articles and meta-analyses. The specific search strategy can be found in **Supplementary Text 1** in **Supplementary Data Sheet 3**.

### 2.2 Inclusion and exclusion criteria

Studies meeting the following criteria were included in this research: (1) Subjects: Patients who were untreated and had activating EGFR mutations (commonly exon 19 deletions or exon 21 L858R mutations), histologically confirmed as advanced or metastatic (stage III-IV) non-squamous NSCLC, regardless of the presence of T790M or other rare EGFR mutations associated with lower sensitivity or resistance; (2) Interventions: Targeted therapies for EGFR mutations, immunotherapies, anti-angiogenic agents used alone or in combination; Comparators: comparisons between chemotherapy alone or other therapies; (3) Study Type: Randomized controlled trials (RCTs); (4) Outcomes: The primary outcomes were progression-free survival (PFS) and overall survival (OS); the secondary outcomes were objective response rate (ORR) and the incidence of grade 3 or higher adverse events (≥3AEs).

Studies were excluded if they met the following criteria: (1) Animal or cell experiments, case reports, scientific study protocols, reviews, letters, editorials, conference papers, etc.; (2) Studies with missing or severely flawed data; (3) Duplicate publications; (4) Full text not available.

Two reviewers (SL and ZMY) independently assessed the titles and abstracts according to these criteria and retrieved relevant full-text articles to screen eligible studies. Disagreements during the screening process were resolved through discussion or by consulting a third reviewer (LXQ).

### 2.3 Data extraction

Two reviewers (SL and ZMY) independently extracted data from the final included studies, including the following aspects: (1) Basic information: first author, year of publication, country; (2) Study characteristics: cancer stage, histological characteristics, race, sample size, age, sex, type of EGFR mutation, interventions, and comparators; (3) Reported outcome measures.

### 2.4 Quality assessment

The quality of the included studies was assessed using the Cochrane Risk of Bias tool (RoB2) (19), evaluating five domains: bias arising from the randomization process, bias due to deviations from intended interventions, bias due to missing outcome data, bias in the measurement of the outcome, and bias in the selection of the reported result. Each study was independently assessed by two researchers, who categorized each domain as "low risk," "high risk," or "some concerns." Disagreements were resolved through discussion or by consulting a third researcher. The assessment results were presented in a risk of bias summary figure.

### 2.5 Statistical analysis

Network meta-analysis was conducted using the R software (version 4.3.2) with the gemtc package (version 1.0-1) and JAGS software, employing the Markov Chain Monte Carlo (MCMC) method within a Bayesian framework (20–22). Four Markov chains were simulated, with an initial value of 2.5, a thinning interval of 1, and 5,000 burn-in iterations to ensure proper annealing. The model was further iterated 20,000 times to achieve convergence. The Deviance Information Criterion (DIC) was used to compare model fit and global consistency (if the absolute difference between the DIC of the consistency and inconsistency models was less than 3, the consistency model was adopted) (23). For networks containing closed loops, the node-splitting method was employed to assess local consistency (24).

Survival outcomes were expressed as hazard ratios (HR), while binary outcomes were presented as relative risk ratios (RR), each accompanied by the corresponding 95% confidence intervals (CIs). A statistically significant difference was considered to be present if the 95% CI did not include the value of 1. A Bayesian random-effects model was used to analyze the efficacy of all treatment options simultaneously. The analysis results included network relationship diagrams, cumulative probability ranking plots, and league tables (25). The Surface Under the Cumulative Ranking Curve (SUCRA) was used as an indicator of cumulative ranking probability, with higher SUCRA values (closer to 100%) indicating better interventions (26). All processes of this network meta-analysis were conducted using Stata 15.0 and R software (version 4.3.2). We conducted a subgroup network meta-analysis (NMA) focusing on the primary survival outcome, progression-free survival (PFS). Subgroup network meta-analyses were performed based on baseline characteristics or subgroup data reported in individual trials, stratified by patient gender, age, and EGFR mutation type. This approach aims to further explore optimal treatment strategies tailored for specific populations.

## 3 Results

### 3.1 Literature search and screening process

**Figure 1:**
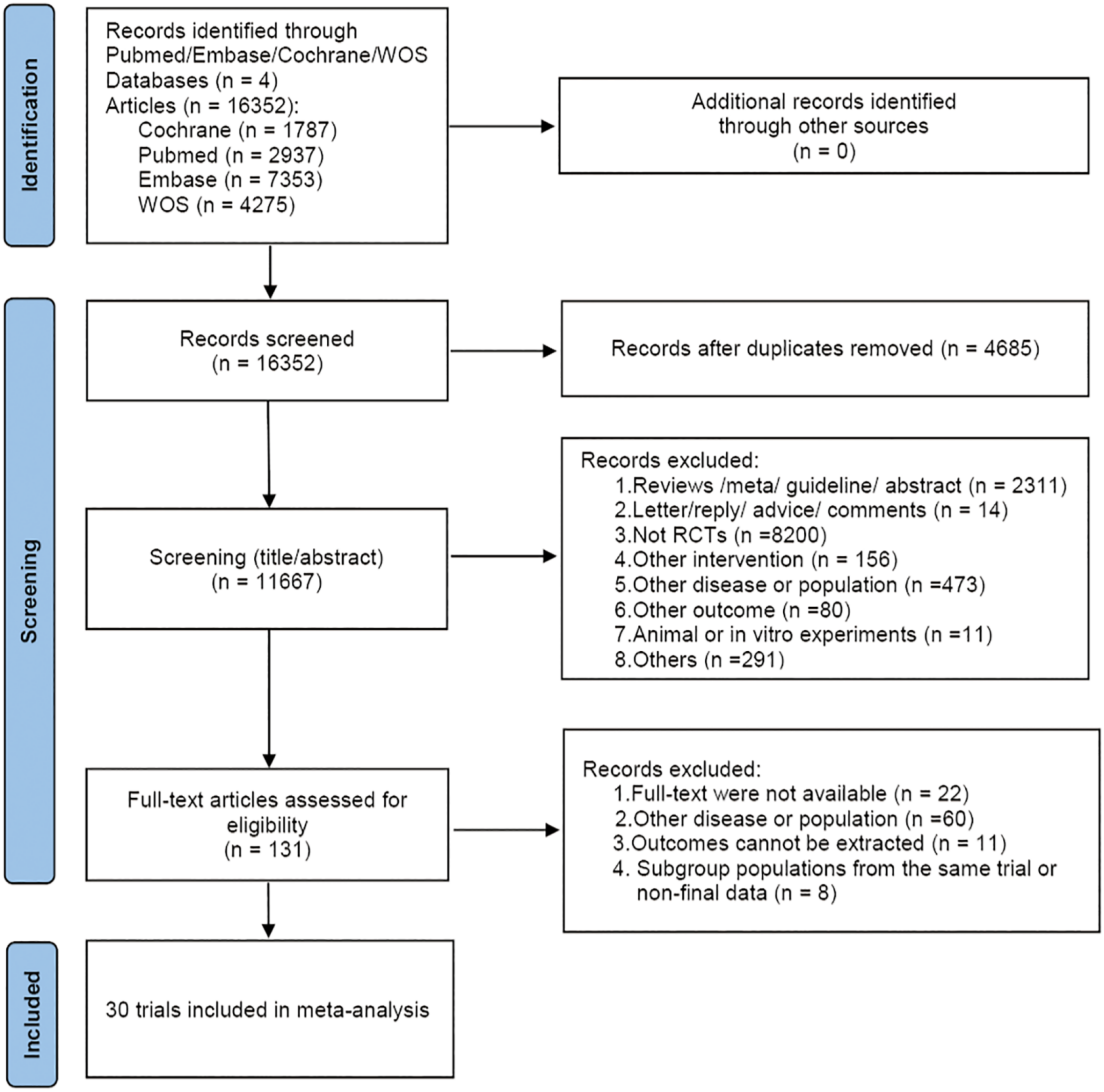
The PRISMA flowchart of the literature search and selection.

A total of 16,352 articles were retrieved. After removing 4,685 duplicates, 11,356 articles were excluded based on the preliminary reading of titles and abstracts. Full-text review was conducted on the remaining 131 articles, and strict inclusion and exclusion criteria were applied, resulting in the inclusion of 30 articles. The specific screening process was illustrated in **Figure 1**.

### 3.2 Basic characteristics of included studies

The 30 included studies (8, 10, 12, 27–52) originated from nine countries: Austria (n=1), China (n=14), France (n=3), India (n=1), Japan (n=3), Korea (n=1), Spain (n=2), The Netherlands (n=1), USA (n=4). These studies involved a total of 8,654 patients, including 3,547 males and 5,107 females, with an age range of 18-87 years. The 8,654 patients in the trials received the following 19 treatments: Chemotherapy; Afatinib; Afatinib + Cetuximab; Apatinib + Gefitinib; Befotertinib; Cetuximab + Chemotherapy; Erlotinib; Erlotinib + Bevacizumab; Erlotinib + Chemotherapy; Gefitinib; Gefitinib + Chemotherapy; Gefitinib + Olaparib; Icotinib; Icotinib + Chemotherapy; Lazertinib; Naquotinib; Osimertinib; Osimertinib + Bevacizumab; Osimertinib + Chemotherapy. Detailed characteristics of the included studies were provided in **Table 1**.

**Table 1:** Characteristics of the included studies.

First Author	Publication Year	Country	Registered ID	Stage	Histologic feature of tumor	Race	Sample size	Age	Sex (male/female)	EGFR mutation type	Treatment		Main Outcomes
											Experimental group	Control group	
Mok et al. (8)	2009	China	NM	IIIB-IV	Adenocarcinoma (all)	East Asia	E: 609 C: 608	E: 57 (24–84) C: 57 (25–84)	E: 125/484 C: 127/481	Exon 19 deletion, exon 21 L858R or T790M and other mutations	Gefitinib 250 mg	Chemotherapy (carboplatin AUC 5 or AUC 6 + paclitaxel 200 mg/m^2^ Q3W)	PFS, OS, ORR
Pirker et al. (27)	2009	Austria	NCT00148798	IIIB-IV	Adenocarcinoma(n=532) Squamous cell carcinoma(n=377) Other(n=216)	White Asian Other	E: 557 C: 568	E: 59 (18–78) C: 60 (20–83)	E: 385/172 C: 405/163	NM	Cetuximab (day 1 400 mg/m², day 8 250 mg/m²) + chemotherapy (cisplatin 80 mg/m² + 25 mg/m² vinorelbine Q3W)	Chemotherapy (cisplatin 80 mg/m² + 25 mg/m² vinorelbine Q3W)	OS
Jänne et al. (28)	2012	USA	NM	IIIB-IV	Adenocarcinoma (n=155) Bronhioloalveolar cancer (n=4) Adenocarcinoma with bronchioloalveolar features(n=22)	White African American Asian Other Unknown	EGFR mutation group: 66	EGFR mutation group: 58(38-79)	EGFR mutation group: 25/41	Exon 19 deletion or L858R mutation in exon 21	Erlotinib 150 mg	Erlotinib 150 mg + chemotherapy (carboplatin AUC 6 + paclitaxel 200 mg/m^2^ Q3W)	PFS, OS
Rosell et al. (10)	2012	Spain	NCT00446225	IIIB-IV	Adenocarcinoma(n=160) Bronchoalveolar adenocarcinoma(n=2) Large-cell carcinoma (n=4) Squamous cell carcinoma (n=1) Other (n=6)	NM	E: 86 C: 87	E: 63·44 ± 10·95 C: 64·15 ± 9·23	E: 28/58 C: 68/19	Exon 19 deletion or L858R mutation in exon 21	Erlotinib 150 mg	Chemotherapy (75 mg/m² cisplatin plus 75 mg/m² docetaxel on day 1 or 75 mg/m² cisplatin on day 1 plus 1250 mg/m² gemcitabine on days 1 and 8 )	PFS
Kenmotsu et al. (29)	2022	Japan	UMIN000030206	IIIB-IV	Non-squamous NSCLC (all)	NM	E: 61 C: 61	E: 67 (59–74) C: 66 (60–74)	E: 24/37 C: 23/38	Exon 19 deletion or L858R mutation in exon 21	Osimertinib 80 mg + bevacizumab 15 mg/kg Q3W	Osimertinib 80 mg Q3W	PFS
WU et al. (30)	2014	China	NCT01121393	IIIB-IV	Adenocarcinoma (n=354)	South-east Asian South Korean Chinese	E: 242 C: 122	E: 58 (49–65) C: 58 (49–62)	E: 87/155 C: 39/83	Exon 19 deletion, L858R mutation in exon 21 and other mutations	Afatinib 40 mg	Chemotherapy (cisplatin 75 mg/m2 + gemcitabine 1000 mg/m²)	PFS, ORR, ≥3 AEs
Yu et al. (31)	2014	China	NCT01769066	IIIB-IV	Adenocarcinoma (n=117)	Asian (Chinese)	E: 58 C: 59	E: 55.3 (36–72) C: 54.9 (33–70)	E: 33/25 C: 25/34	Mutant (19/21 exon) Wild type Unknown 20 exon mutation 20 and 21 exon mutation	Chemotherapy + gefitinib 250 mg	Chemotherapy (pemetrexed 500 mg/m^2^) and either cisplatin (75 mg/ m^2^ d1) or carboplatin (AUC = 5 d1)	PFS, ORR
Yang et al. (32)	2015	China	NCT00949650 NCT01121393	IIIB-IV	Adenocarcinoma (all)	NA	LUX-Lung 3 E: 230 C: 115 LUX-Lung 6 E: 242 C: 122	LUX-Lung 3 E: 62 (28–86) C: 61 (31–83) LUX-Lung 6 E: 58 (29–79) C: 58 (27–76)	LUX-Lung 3 E: 83/147 C: 38/77 LUX-Lung 6 E: 87/155 C: 39/83	19 deletion Leu858Arg mutation Uncommon mutations	LUX-Lung 3 Afatinib 40 mg LUX-Lung 6 Afatinib	LUX-Lung 3 Chemotherapy(pemetrexed 500 mg/m^2^ + cisplatin 75 mg/m² Q3W) LUX-Lung 6 Chemotherapy(gemcitabine 1000 mg/m² + cisplatin 75 mg/m²)	OS, ORR, ≥3 AEs
An et al. (33)	2016	China	NM	IIIB-IV	Adenocarcinoma (n=90)	Asian (Chinese)	E: 45 C: 45	E: 66.89 ± 12.46 C: 65.72 ± 13.02	E: 25/20 C: 25/20	Exon 19 deletion or L858R mutation in exon 21	Gefitinib 250 mg + placebo	Pemetrexed 500 mg/m^2^ + Gefitinib 250 mg	PFS, OS, ORR, ≥3 AEs
Yang et al. (34)	2016	China	NCT01017874	IIIB-IV	Adenocarcinoma (n=229) Non adenocarcinoma (n=7)	EAST Asian	E: 118 C: 118	E: 59 (24–81) C: 59 (31–79)	E: 30/88 C: 29/89	Wild-type EGFR EGFR mutation-positive EGFR mutation status unknown	Chemotherapy (pemetrexed 500 mg/m^2^ + cisplatin 75 mg/ m^2^ Q3W) + gefitinib 250 mg	Gefitinib 250 mg	OS
Han et al. (35)	2017	China	NCT02148380	IIIB-IV	Adenocarcinoma(all)	NM	E1: 40 E2: 40 C: 41	E1: <65: 27; ≥65: 13 E2: <65: 31; ≥65: 9 C: <65: 27; ≥65: 14	E1: 15/25 E2: 17/23 C: 18/23	Exon 19 deletion or L858R mutation in exon 21	E1: Gefitinib 250 mg + chemotherapy (carboplatin AUC5 + paclitaxel 500 mg/m2 Q4W) E2: Chemotherapy (carboplatin AUC 5 + paclitaxel 500 mg/m2 Q4W )	Gefitinib 250 mg	PFS, OS, ORR, ≥3 AEs
Patil et al. (36)	2017	India	CTRI/2015/08/006113	IIIB-IV	Adenocarcinoma(all)	NM	E: 145 C: 145	E: 54.44 C: 53.12	E: 67/78 C: 97/48	Mutations in exons 18, 19 or 21	Gefitinib 250 mg	Chemotherapy (carboplatin AUC 5 + Pemetrexed 500 mg/m^2^ Q3W)	PFS, OS, ORR
Shi et al. (37)	2017	China	NM	IIIB-IV	Adenocarcinoma(all)	NM	E: 148 C: 137	E: 56 (35.3–73.7) C: 56 (30.5–76.9)	E: 43/105 C: 42/95	Exon 19 deletion or L858R mutation in exon 21	Icotinib 125 mg	Chemotherapy(pemetrexed 500 mg/m^2^ + cisplatin 75 mg/m² Q3W)	PFS, OS
Herbst et al. (38)	2018	USA	NCT00946712	IV	Adenocarcinoma(n=819) Squamous cell carcinoma(n=321) Other(n=173)	NM	EGFR FISH-positive E: 199 C: 201	EGFR FISH-positive E: 62 (37–80) C: 64 (34–84)	EGFR FISH-positive E: 125/74 C: 115/86	NM	Cetuximab 400 mg/m² + chemotherapy + Bevacizumab*	Chemotherapy (carboplatin AUC 6 + paclitaxel 200 mg/m^2^ Q3W) + Bevacizumab*	PFS, OS, ORR
Soria et al. (12)	2018	France	NCT02296125	Locally advanced or metastatic	Adenocarcinoma(n=547) Other(n=9)	White Asian Other	E: 279 C: 277	E: 64 (26–85) C: 64 (35–93)	E: 101/178 C: 105/172	Exon 19 deletion or L858R mutation in exon 21	Osimertinib 80mg	Standard EGFR-TKI (gefitinib 250 mg or erlotinib 150 mg)	PFS, OS, ORR, ≥3 AEs
Kelly et al. (39)	2019	USA	NCT02588261	IIIB-IV	Adenocarcinoma(all)	NM	E: 267 C: 263	E: 68 (32–88) C: 67 (23–89)	E: 96/171 C: 110/153	Exon 19 deletion, exon 21 L858R and T790M	ASP8273 300 mg	Standard EGFR-TKI (gefitinib 250 mg or erlotinib 150 mg)	PFS, ORR, ≥3 AEs
Stinchcombe et al. (40)	2019	USA	NCT01532089	IV	Non-squamous NSCLC (all)	White African American Asian	E: 45 C: 43	E: 63 (47-84) C: 65 (31-84)	E: 14/31 C: 12/31	Exon 19 deletion or L858R mutation in exon 21	Erlotinib 150 mg Q3W	Erlotinib 150 mg + bevacizumab 15 mg Q3W	PFS, OS
Xu et al. (41)	2019	China	NCT0203160	IIIA-IV	Adenocarcinoma(all)	NM	E: 90 C: 89	E: <65: 61; ≥65: 29 C: <65: 57; ≥65: 32	E: 33/57 C: 23/66	Mutations in exons 18, 19 or 21	Icotinib 125 mg + chemotherapy (carboplatin AUC 5 + pemetrexed 500 mg/m^2^ Q3W)	Icotinib 125 mg	PFS, OS, ORR
Ye et al.	2019	China	NM	III-IV	Adenocarcinoma(all)	NM	E: 49 C: 49	E: <75: 29; ≥75: 20 C: <75: 267; ≥75: 23	E: 19/30 C: 21/28	NM	Icotinib 125 mg Q4W	Chemotherapy (cisplatin 25 mg/m^2^ + pemetrexed 500 mg/m^2^ Q3W)	PFS, ORR
Garcia-Campelo et al. (42)	2020	Spain	NCT01513174	IV	Adenocarcinoma(all)	NM	E: 91 C: 91	E: 68 (36- 85) C: 65 (39-85)	E: 34/57 C: 25/66	Exon 19 deletion L858R Exon 18 Exon 20 Unknown	Gefitinib 250 mg	Gefitinib 250 mg + olaparib 200 mg	PFS, OS, ORR, ≥3 AEs
Cortot et al. (43)	2021	France	NCT02716311	III-IV	Non-squamous NSCLC (all)	NM	E: 59 C: 58	E: 68.1(34-86.2) C: 63.8(41.7- 84.3)	E: 16/43 C: 17/41	Deletion exon 19 Mutation G719X exon 18 Mutation L858R exon 21 Mutation L861Q	Afatinib 40 mg Q4W	Afatinib 40 mg + cetuximab 250 mg/m^2^ Q4W	PFS, OS, ORR, ≥3 AEs
Zhao et al. (44)	2021	China	NCT02824458	IIIB-IV	Non-squamous NSCLC (all)	NM	E: 157 C: 156	E: 57 (51,65) C: 60 (51,65)	E: 66/91 C: 62/94	Exon 19 deletion or L858R mutation in exon 21	Apatinib 500 mg + gefitinib 250 mg Q4W	Placebo + gefitinib 250 mg Q4W	PFS, OS, ORR, ≥3 AEs
Gijtenbeek et al. (45)	2022	The Netherlands	NM	IV	Non-squamous NSCLC (all)	NM	E: 11 C: 11	E: 60 (58–64) C: 67 (62–68)	E: 5/6 C: 5/6	Mutations in exons 18, 19 or 21	Erlotinib 150 mg + chemotherapy (pemetrexed 500 mg/m^2^ + cisplatin 75 mg/m² Q3W)	Erlotinib 150 mg	PFS, OS
Cho et al. (46)	2023	Korea	NCT04248829	Locally advanced or metastatic	Adenocarcinoma(all)	Asian Non-Asian	E: 196 C: 197	E: 67 (31-87) C: 64 (25-86)	E: 64/132 C: 78/119	Exon 19 deletion or L858R mutation in exon 21	Lazertinib 240 mg	Gefitinib 250 mg	PFS, OS, ORR, ≥3 AEs
Lu et al. (47)	2023	China	NCT04206072	IIIB-IV	Adenocarcinoma(all)	Asian (Chinese)	E: 182 C: 180	E: 60 (53–66) C: 58 (53–65)	E: 72/110 C: 72/108	Exon 19 deletion or L858R mutation in exon 21	Befotertinib 75mg	Icotinib 125 mg	PFS, OS, ORR
Planchard et al. (48)	2023	France	NCT04035486	Advanced	Non-squamous NSCLC (all)	Asian White American Indian orAlaska NMtive Black Other	E: 279 C: 278	E: 61 (26–83) C: 62 (30–85)	E: 106/173 C: 109/169	Exon 19 deletion L858R mutation Both exon 19 deletion and L858R mutation Unknown	Osimertinib 80mg + chemotherapy(pemetrexed 500 mg/m^2^ + cisplatin 75 mg/m² OR carboplatin Q3W)	Osimertinib 80mg	PFS, OS, ORR, ≥3 AEs
Zhou et al. (49)	2011	China	NCT00874419	IIIB-IV	Adenocarcinoma(n=134) Non-adenocarcinoma(n=20)	Asian (Chinese)	E: 82 C: 72	E: 57 (31–74) C: 59 (36–78)	E: 34/48 C: 29/43	Exon 19 deletion or L858R mutation in exon 21	Erlotinib 150 mg	Chemotherapy (carboplatin AUC 5 + gemcitabine 1000 mg/m² Q3W)	PFS
Zhou et al. (50)	2015	China	NCT00874419	IIIB-IV	Adenocarcinoma(n=134) Non-adenocarcinoma(n=20)	Asian (Chinese)	E: 82 C: 72	E: 57 (31–74) C: 59 (36–78)	E: 34/48 C: 29/43	Exon 19 deletion or L858R mutation in exon 21	Erlotinib 150 mg	Chemotherapy (carboplatin AUC 5 + gemcitabine 1000 mg/m² Q3W)	OS
Seto et al. (51)	2014	Japan	JapicCTI-111390	IIIB-IV	Adenocarcinoma (n=150) Large-cell carcinomaa(n=1) Adenosquamous carcinomaa(n=1)	NM	E: 75 C: 77	E: 67.0 (59–73) C: 67.0 (60–73)	E: 30/45 C: 26/51	Exon 19 deletion or L858R mutation in exon 21	Erlotinib 150 mg Q3W	Erlotinib 150 mg + bevacizumab 15 mg/kg Q3W	PFS
Yamamoto et al. (52)	2021	Japan	JapicCTI-111390	IIIB-IV	Adenocarcinoma (n=150) Large-cell carcinomaa(n=1) Adenosquamous carcinomaa(n=1)	NM	E: 75 C: 77	E: 67.0 (59–73) C: 67.0 (60–73)	E: 30/45 C: 26/51	Exon 19 deletion or L858R mutation in exon 21	Erlotinib 150 mg Q3W	Erlotinib 150 mg + bevacizumab 15 mg/kg Q3W	OS

NM, not mentioned; NA, not applicable; Data are expressed as Mean±SD, Median(IQR) or Median (range). E, Experimental group; C, Control group; PFS, progression-free survival; OS, overall survival; ORR, Objective response rate; 3AEs, the incidence of grade 3 or higher adverse events; *, yes or no; Q3W, every 3 weeks; Q4W, every 4 weeks.

### 3.3 Methodological quality assessment of included studies

**Figure 2:**
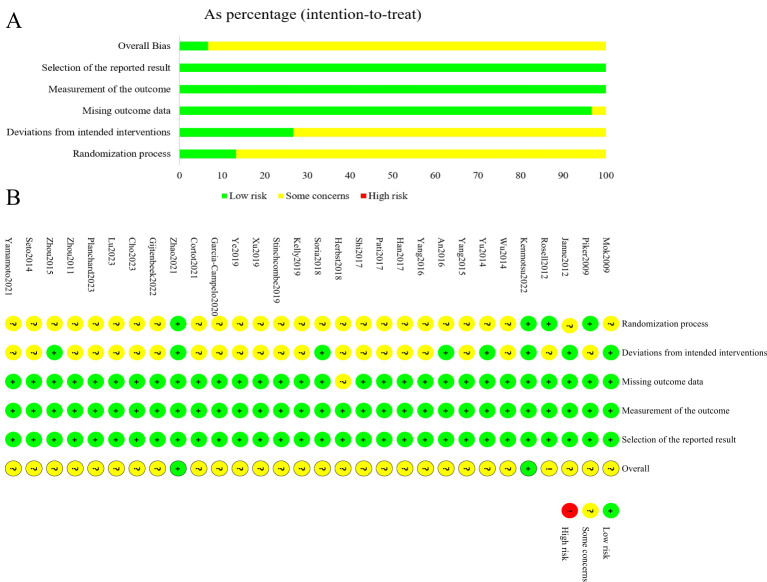
Summary results on risk of bias (using RoB2) of including RCTs. **(A)**, Percent of studies with categories for risk of bias; **(B)**, Summary for the risk of bias in each study.

The results of the risk of bias assessment for the 30 included studies were shown in **Figure 2**. In terms of bias arising from the randomization process, 27 studies were assessed as potentially having a risk due to the lack of random allocation or lack of allocation concealment, while the remaining 3 studies were deemed low risk. Regarding bias due to deviations from intended interventions, 22 studies were assessed as potentially having a risk because they were open-label and blinding was not feasible, while the remaining 8 studies were assessed as low risk. Except for one study assessed as potentially having a risk due to missing outcome data, the remaining 29 studies were assessed as low risk for both missing outcome data and measurement outcomes. No selective reporting was detected in any of the studies, and this was assessed as low risk. Overall, the risk of bias in the included literature was moderate.

### 3.4 Network meta-analysis results

#### 3.4.1 Network evidence diagram

**Figure 3:**
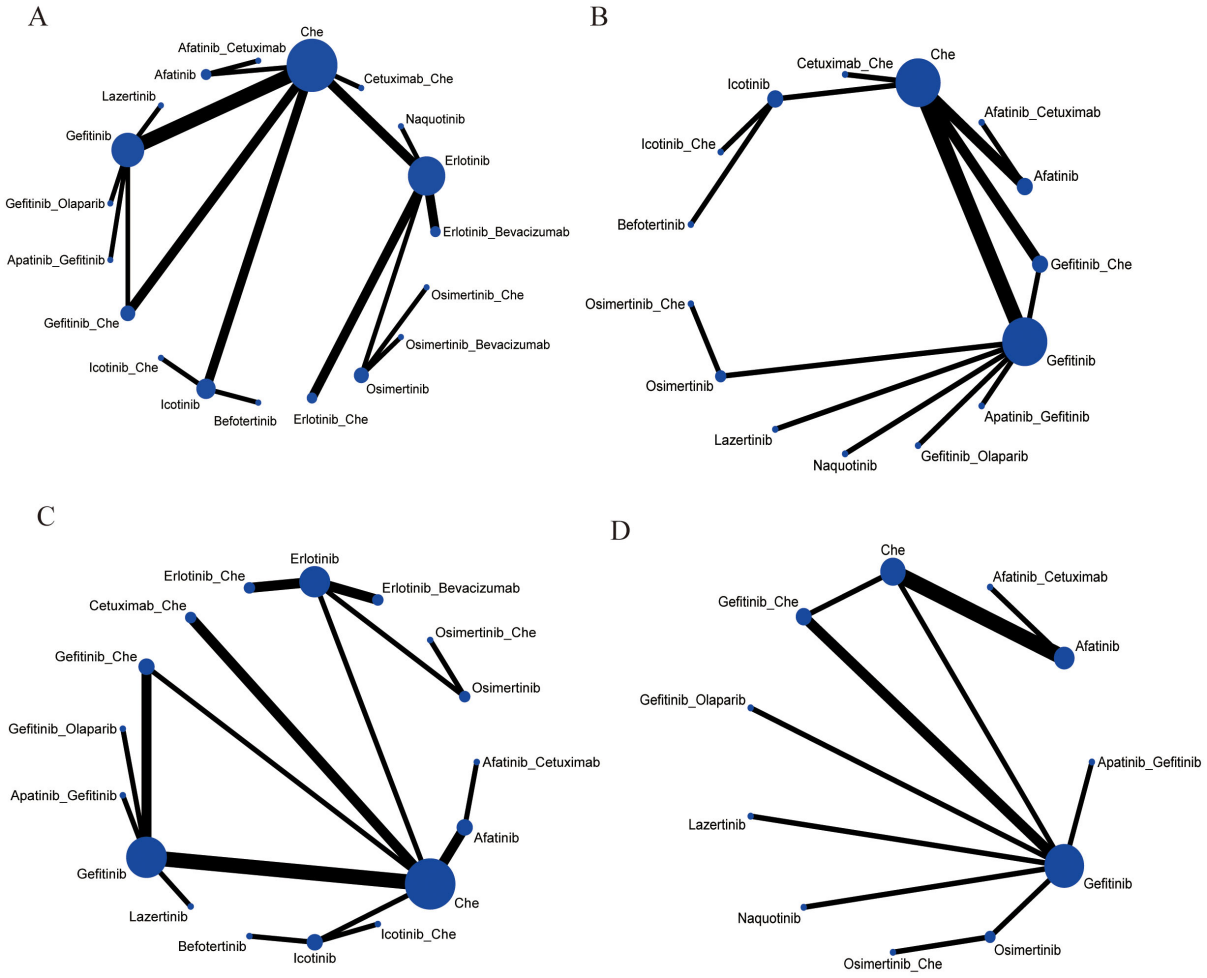
Network meta-analysis map concerning the efficacy and safety of first-line treatments in patients with EGFR mutation-positive advanced or metastatic non-squamous non-small cell lung cancer on **(A)** PFS, **(B)** OS, **(C)** ORR, **(D)** ≥3 AEs. The size of the nodes relates to the number of participants in that intervention type, and the thickness of lines between the interventions relates to the number of studies for that comparison. Che, Chemotherapy.

The 30 included studies covered 19 different interventions: Chemotherapy; Afatinib; Afatinib + Cetuximab; Apatinib + Gefitinib; Befotertinib; Cetuximab + Chemotherapy; Erlotinib; Erlotinib + Bevacizumab; Erlotinib + Chemotherapy; Gefitinib; Gefitinib + Chemotherapy; Gefitinib + Olaparib; Icotinib; Icotinib + Chemotherapy; Lazertinib; Naquotinib; Osimertinib; Osimertinib + Bevacizumab; Osimertinib + Chemotherapy. The network structure of different interventions for each outcome indicator was shown in **Figure 3**. In the diagram, the thickness of the lines is proportional to the number of studies comparing the two interventions, and the diameter of the circles is proportional to the number of participants included for each intervention.

#### 3.4.2 Progression-free survival

**Figure 4:**
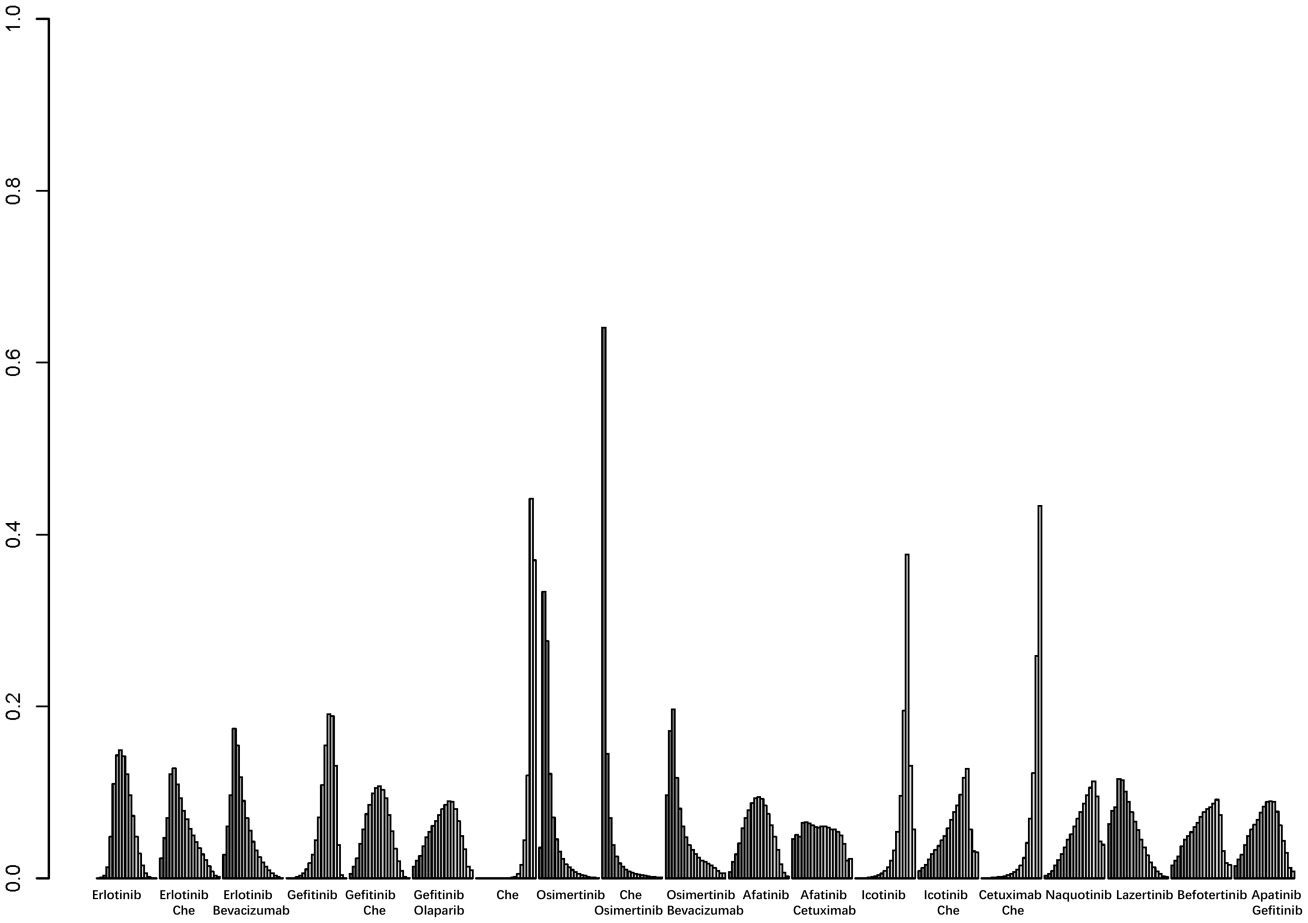
Result of probability ranking for optimal PFS among different intervention measures. The horizontal axis represents the various intervention measures, and the vertical axis indicates the probability of ranking for each intervention measure. Each bar graph shows the probability of different intervention measures being ranked from first position to last position.

A total of 25 studies reported PFS outcomes for 19 interventions: Chemotherapy; Afatinib; Afatinib + Cetuximab; Apatinib + Gefitinib; Befotertinib; Cetuximab + Chemotherapy; Erlotinib; Erlotinib + Bevacizumab; Erlotinib + Chemotherapy; Gefitinib; Gefitinib + Chemotherapy; Gefitinib + Olaparib; Icotinib; Icotinib + Chemotherapy; Lazertinib; Naquotinib; Osimertinib; Osimertinib + Bevacizumab; Osimertinib + Chemotherapy (8, 10, 28–31, 33, 35–49, 51). The network structure for different interventions was shown in **Figure 3A**. The network meta-analysis results indicated that Afatinib (HR=0.28, 95% CI: 0.1, 0.79), Apatinib + Gefitinib (HR=0.3, 95% CI: 0.09, 0.91), Erlotinib (HR=0.25, 95% CI: 0.12, 0.54), Erlotinib + Bevacizumab (HR=0.18, 95% CI: 0.06, 0.53), Erlotinib + Chemotherapy (HR=0.21, 95% CI: 0.06, 0.64), Gefitinib (HR=0.43, 95% CI: 0.23, 0.71), Gefitinib + Chemotherapy (HR=0.27, 95% CI: 0.13, 0.58), Gefitinib + Olaparib (HR=0.31, 95% CI: 0.09, 0.95), Lazertinib (HR=0.19, 95% CI: 0.06, 0.58), Osimertinib + Chemotherapy (HR=0.07, 95% CI: 0.01, 0.37), Osimertinib (HR=0.12, 95% CI: 0.03, 0.41), and Osimertinib + Bevacizumab (HR=0.14, 95% CI: 0.03, 0.74) were significantly better than chemotherapy in improving PFS. Furthermore, Erlotinib, Erlotinib + Bevacizumab, Erlotinib + Chemotherapy, Gefitinib + Chemotherapy, Lazertinib, Osimertinib, Osimertinib + Bevacizumab, and Osimertinib + Chemotherapy were significantly better than Cetuximab + Chemotherapy (HR=0.26, 95% CI: 0.07, 0.89; HR=0.18, 95% CI: 0.04, 0.79; HR=0.21, 95% CI: 0.04, 0.94; HR=0.28, 95% CI: 0.08, 0.96; HR=0.19, 95% CI: 0.04, 0.85; HR=0.12, 95% CI: 0.02, 0.59; HR=0.14, 95% CI: 0.02, 0.98; HR=0.07, 95% CI: 0.01, 0.5, respectively). No significant differences were found among the other pairwise comparisons (**Table 2**). Based on cumulative probability results, Osimertinib + Chemotherapy (SUCRAs: 93.4%), Osimertinib (SUCRAs: 84.61%), and Osimertinib + Bevacizumab (SUCRAs: 76.31%) might be the top three measures for improving PFS (**Figure 4**).

**Table 2:** The PFS league table.

Lazertinib	Naquotinib	Osimertinib	Osimertinib_Bevacizumab	Osimertinib_Che
0.68(0.13,3.07)	1.46(0.28,7.57)	0.41(0.08,2.09)	0.49(0.07,3.53)	0.26(0.04,1.73)
0.72(0.1,4.69)	1.53(0.2,11.4)	0.44(0.06,3.16)	0.52(0.05,4.97)	0.27(0.03,2.5)
0.63(0.15,2.66)	1.35(0.25,8.21)	0.38(0.07,2.27)	0.46(0.06,3.75)	0.24(0.03,1.86)
0.59(0.09,3.11)	1.25(0.2,7.62)	0.36(0.06,2.12)	0.42(0.05,3.44)	0.22(0.03,1.72)
**0.19(0.04,0.85)**	0.41(0.08,2.12)	**0.12(0.02,0.59)**	**0.14(0.02,0.98)**	**0.07(0.01,0.5)**
**0.19(0.06,0.58)**	0.41(0.11,1.49)	**0.12(0.03,0.41)**	**0.14(0.03,0.74)**	**0.07(0.01,0.37)**
0.75(0.18,2.88)	1.61(0.56,4.64)	0.46(0.17,1.26)	0.55(0.12,2.42)	0.28(0.07,1.2)
1.06(0.21,4.89)	2.27(0.62,8.28)	0.65(0.18,2.27)	0.77(0.14,4.09)	0.4(0.08,2.03)
0.93(0.17,4.58)	1.99(0.51,7.83)	0.57(0.15,2.14)	0.67(0.12,3.82)	0.35(0.07,1.87)
0.45(0.16,1.25)	0.96(0.25,4.15)	0.27(0.07,1.15)	0.32(0.06,2.02)	0.17(0.03,0.99)
0.7(0.18,2.46)	1.5(0.34,6.71)	0.43(0.1,1.87)	0.51(0.08,3.22)	0.26(0.04,1.6)
0.62(0.14,2.65)	1.32(0.24,8.04)	0.38(0.07,2.24)	0.45(0.06,3.66)	0.23(0.03,1.85)
0.29(0.07,1.08)	0.61(0.13,2.73)	0.17(0.04,0.75)	0.21(0.03,1.29)	0.11(0.02,0.64)
0.49(0.08,2.59)	1.04(0.16,6.41)	0.3(0.05,1.78)	0.35(0.04,2.89)	0.18(0.02,1.46)
Lazertinib	2.13(0.4,12.83)	0.61(0.11,3.59)	0.72(0.1,5.88)	0.37(0.05,2.95)
0.47(0.08,2.52)	Naquotinib	0.28(0.07,1.23)	0.34(0.05,2.11)	0.18(0.03,1.05)
1.65(0.28,8.7)	3.51(0.81,15.18)	Osimertinib	1.19(0.4,3.55)	0.62(0.22,1.71)
1.39(0.17,10.12)	2.95(0.47,18.4)	0.84(0.28,2.53)	Osimertinib_Bevacizumab	0.52(0.12,2.34)
2.67(0.34,18.44)	5.68(0.95,33.74)	1.62(0.58,4.47)	1.92(0.43,8.63)	Osimertinib_Che

Estimates are presented as column versus row for the network meta-analyses to make network meta-analysis results directly comparable. Effect estimates are presented as pooled HR with 95% CIs. Che, Chemotherapy. Bold values indicate statistical significance (P<0.05).

#### 3.4.3 Overall survival

**Figure 5:**
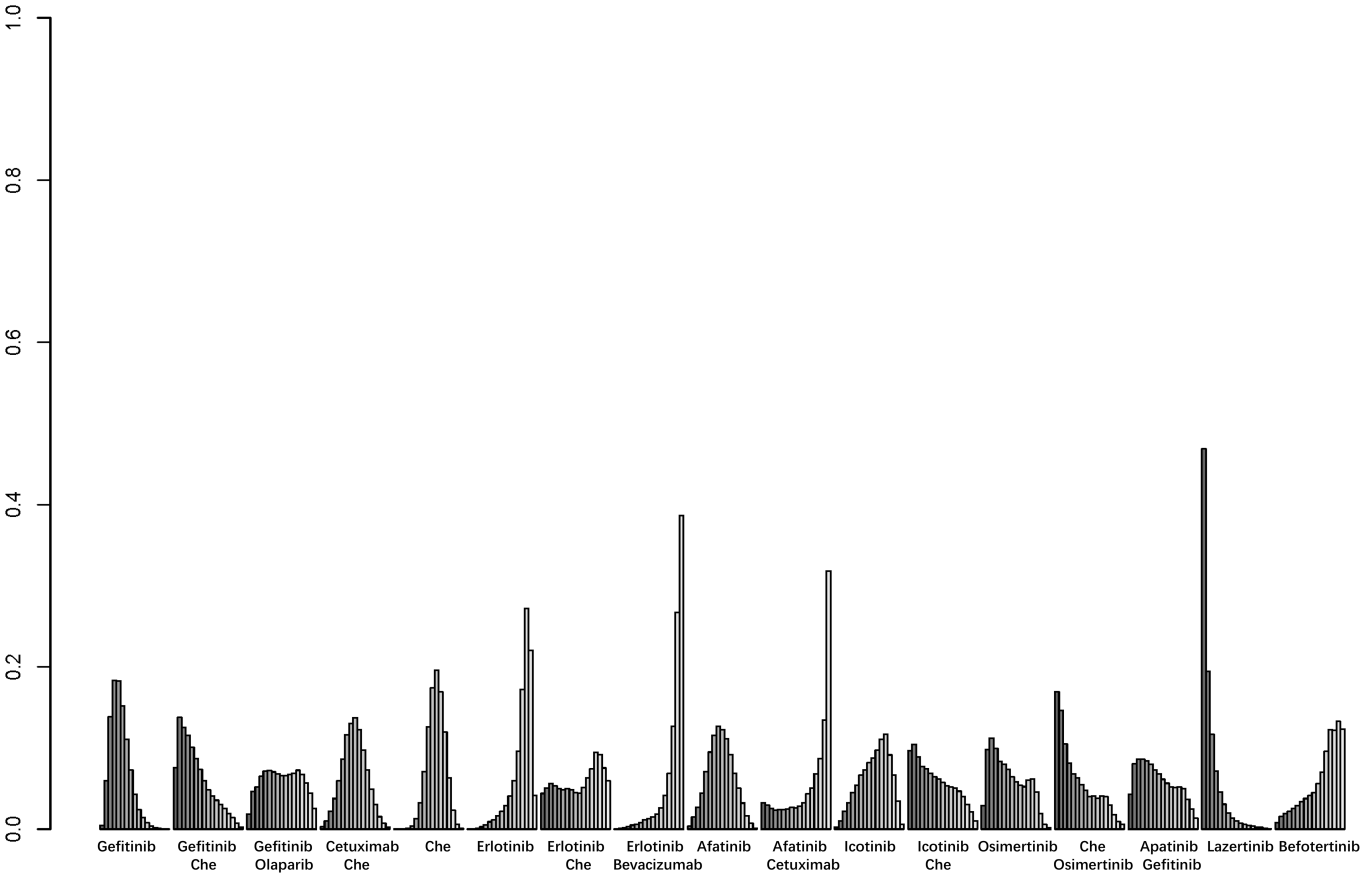
Result of probability ranking for optimal OS among different intervention measures.

A total of 22 studies reported OS outcomes for 17 interventions: Chemotherapy; Afatinib; Afatinib + Cetuximab; Apatinib + Gefitinib; Befotertinib; Cetuximab + Chemotherapy; Erlotinib; Erlotinib + Bevacizumab; Erlotinib + Chemotherapy; Gefitinib; Gefitinib + Chemotherapy; Gefitinib + Olaparib; Icotinib; Icotinib + Chemotherapy; Lazertinib; Osimertinib; Osimertinib + Chemotherapy (8, 12, 27, 28, 32, 33, 35–38, 40–45, 47, 48, 50–52). The network structure for different interventions is shown in **Figure 3B**. The network meta-analysis results indicated that Lazertinib (HR=0.56, 95% CI: 0.32, 0.99) was significantly better than chemotherapy in improving OS. Lazertinib (HR=0.44, 95% CI: 0.21, 0.9) and Osimertinib (HR=0.63, 95% CI: 0.4, 0.98) were significantly better than Erlotinib. Additionally, Lazertinib (HR=0.37, 95% CI: 0.15, 0.85) and Osimertinib (HR=0.53, 95% CI: 0.28, 0.98) were significantly better than Erlotinib + Bevacizumab. No significant differences were found among the other pairwise comparisons (**Supplementary Table 1**). Based on cumulative probability results, Lazertinib (SUCRAs: 89.72%), Gefitinib (SUCRAs: 72.07%), and Osimertinib + Chemotherapy (SUCRAs: 70.74%) might be the top three measures for improving OS (**Figure 5**).

#### 3.4.4 Objective response rate

**Figure 6:**
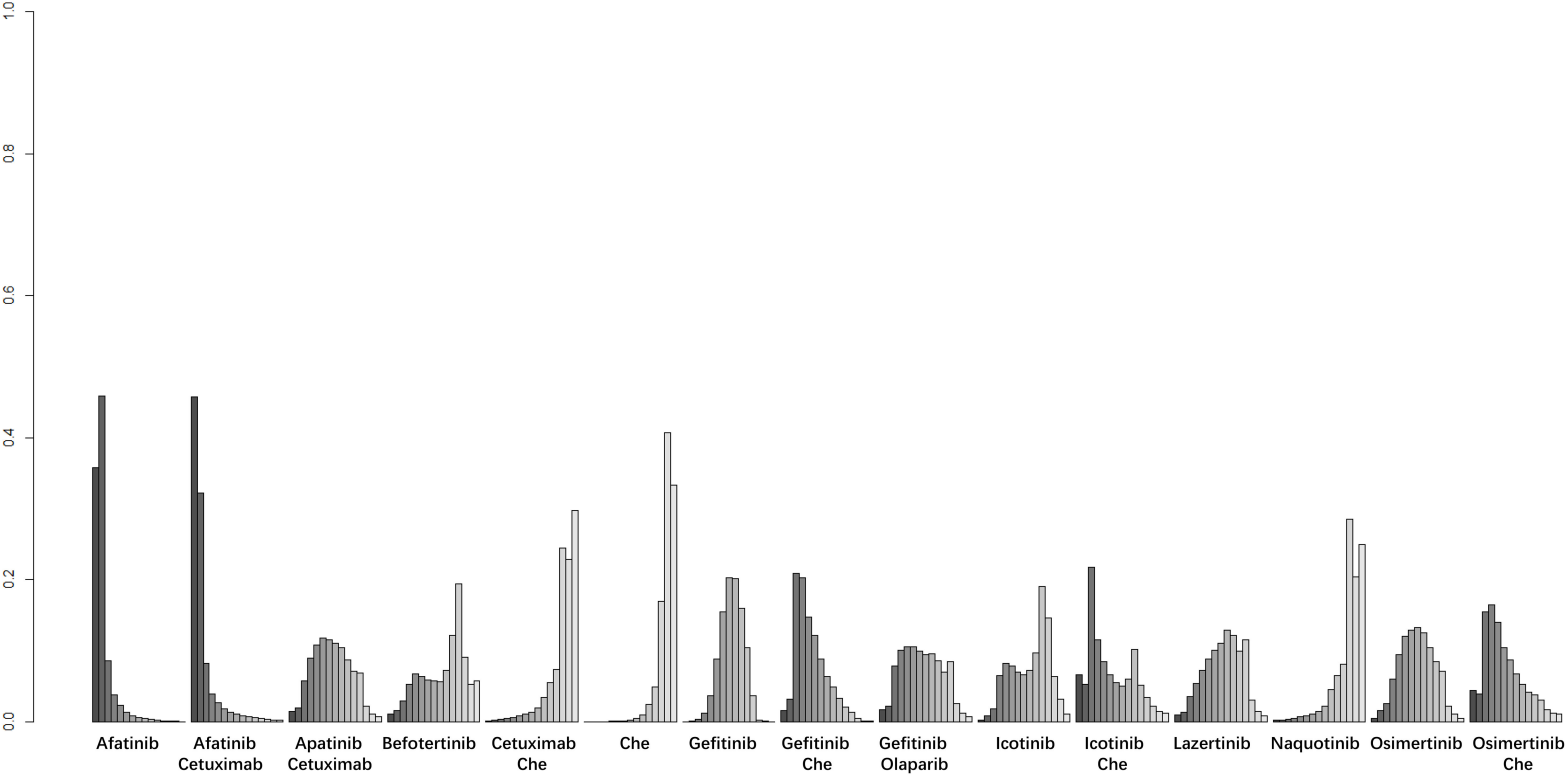
Result of probability ranking for optimal ORR among different intervention measures.

A total of 18 studies were included for analysis (8, 12, 30–33, 35–39, 41–44, 46–48), involving the following 15 interventions: Afatinib, Afatinib + Cetuximab, Apatinib + Gefitinib, Befotertinib, Cetuximab + Chemotherapy, Chemotherapy, Gefitinib, Gefitinib + Chemotherapy, Gefitinib + Olaparib, Icotinib, Icotinib + Chemotherapy, Lazertinib, Naquotinib, Osimertinib, Osimertinib + Chemotherapy. The network structure for different interventions was shown in **Figure 3C**. The network meta-analysis results showed that Afatinib (HR=2.73, 95% CI: 1.88, 3.98), Afatinib + Cetuximab (HR=2.77, 95% CI: 1.52, 5.02), Apatinib + Gefitinib (HR=1.65, 95% CI: 1.02, 2.85), Gefitinib (HR=1.58, 95% CI: 1.24, 2.12), and Gefitinib + Chemotherapy (HR=1.86, 95% CI: 1.35, 2.7) had higher ORR compared to chemotherapy. Afatinib and Afatinib + Cetuximab also showed higher ORR compared to Cetuximab + Chemotherapy (HR=2.6, 95% CI: 1.41, 4.83; HR=2.63, 95% CI: 1.22, 5.69; respectively). Afatinib and Afatinib + Cetuximab showed higher ORR compared to Naquotinib (HR=2.53, 95% CI: 1.28, 4.72; HR=2.56, 95% CI: 1.1, 5.53; respectively). and Afatinib showed significantly higher ORR compared to Cetuximab + Chemotherapy (HR=2.6, 95% CI: 1.41, 4.83). Other pairwise intervention differences were not statistically significant (**Supplementary Table 2**). Based on cumulative probability results, Afatinib (SUCRAs: 92.27%), Afatinib + Cetuximab (SUCRAs: 90.95%), and Gefitinib + Chemotherapy (SUCRAs: 69.05%) might be the top three measures for ORR (**Figure 6**).

#### 3.4.5 Grade ≥3 adverse events

**Figure 7:**
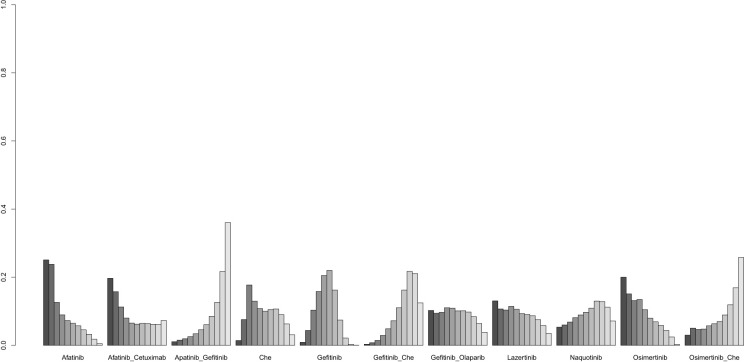
Result of probability ranking for optimal ≥ 3 AEs among different intervention measures.

A total of 11 studies were included for analysis (12, 30, 32, 33, 35, 39, 42, 43, 46, 48), involving the following 11 interventions: Afatinib, Afatinib + Cetuximab, Apatinib + Gefitinib, Chemotherapy, Gefitinib, Gefitinib + Chemotherapy, Gefitinib + Olaparib, Lazertinib, Naquotinib, Osimertinib, Osimertinib + Chemotherapy. The network structure for different interventions was shown in **Figure 3D**. The network meta-analysis results indicated no statistically significant differences in the incidence of grade ≥3 adverse events between any treatment and chemotherapy or other treatments (**Table 3**). Based on cumulative probability results, Afatinib (SUCRAs: 74.93%), Osimertinib (SUCRAs: 69.42%), and Afatinib + Cetuximab (SUCRAs: 61.87%) might be the top three measures in terms of safety (**Figure 7**).

**Table 3:** The ≥3 AEs league table.

Afatinib_Cetuximab	Apatinib_Gefitinib	Chemotherapy	Gefitinib	Gefitinib_Chemotherapy	Gefitinib_Olaparib	Lazertinib	Naquotinib	Osimertinib	Osimertinib_Chemotherapy
1.24 (0.31, 4.98)	3.56 (0.37, 29.52)	1.54 (0.7, 3.36)	1.56 (0.27, 8.19)	2.71 (0.53, 14.35)	1.57 (0.17, 13.1)	1.46 (0.15, 12.35)	1.97 (0.21, 16.26)	1.18 (0.13, 9.7)	2.76 (0.2, 33.17)
Afatinib_Cetuximab	2.87 (0.2, 35.02)	1.24 (0.25, 6.13)	1.26 (0.13, 10.68)	2.18 (0.25, 19.23)	1.26 (0.09, 15.53)	1.18 (0.08, 14.85)	1.59 (0.11, 19.43)	0.95 (0.07, 11.77)	2.23 (0.11, 38.35)
0.35 (0.03, 4.95)	Apatinib_Gefitinib	0.43 (0.06, 3.55)	0.44 (0.12, 1.7)	0.76 (0.15, 4.46)	0.44 (0.07, 3)	0.41 (0.06, 2.83)	0.55 (0.08, 3.76)	0.33 (0.05, 2.23)	0.78 (0.08, 8.12)
0.81 (0.16, 4)	2.3 (0.28, 16.57)	Chemotherapy	1.01 (0.21, 4.45)	1.76 (0.42, 7.77)	1.02 (0.13, 7.32)	0.95 (0.11, 7)	1.27 (0.16, 9.16)	0.76 (0.09, 5.47)	1.79 (0.15, 18.95)
0.79 (0.09, 7.65)	2.26 (0.59, 8.62)	0.99 (0.22, 4.68)	Gefitinib	1.73 (0.67, 5.17)	1 (0.26, 3.86)	0.93 (0.23, 3.72)	1.26 (0.33, 4.8)	0.75 (0.2, 2.86)	1.76 (0.26, 11.69)
0.46 (0.05, 3.93)	1.31 (0.22, 6.6)	0.57 (0.13, 2.4)	0.58 (0.19, 1.5)	Gefitinib_Chemotherapy	0.58 (0.1, 2.94)	0.54 (0.09, 2.82)	0.73 (0.13, 3.65)	0.43 (0.07, 2.17)	1.02 (0.11, 8.25)
0.79 (0.06, 11.29)	2.27 (0.33, 15.36)	0.98 (0.14, 7.99)	1 (0.26, 3.89)	1.73 (0.34, 10.14)	Gefitinib_Olaparib	0.93 (0.13, 6.44)	1.26 (0.19, 8.43)	0.75 (0.11, 5)	1.76 (0.17, 18.16)
0.85 (0.07, 12.12)	2.43 (0.35, 16.69)	1.06 (0.14, 8.75)	1.07 (0.27, 4.28)	1.86 (0.35, 11.11)	1.07 (0.16, 7.42)	Lazertinib	1.35 (0.19, 9.25)	0.81 (0.12, 5.52)	1.89 (0.18, 19.87)
0.63 (0.05, 8.86)	1.8 (0.27, 11.98)	0.79 (0.11, 6.25)	0.8 (0.21, 3.06)	1.38 (0.27, 7.9)	0.79 (0.12, 5.34)	0.74 (0.11, 5.14)	Naquotinib	0.6 (0.09, 4)	1.4 (0.14, 14.4)
1.06 (0.08, 14.96)	3.01 (0.45, 20.1)	1.31 (0.18, 10.56)	1.33 (0.35, 5.07)	2.3 (0.46, 13.38)	1.33 (0.2, 8.91)	1.24 (0.18, 8.52)	1.67 (0.25, 11.12)	Osimertinib	2.34 (0.61, 9.05)
0.45 (0.03, 8.84)	1.29 (0.12, 13.16)	0.56 (0.05, 6.68)	0.57 (0.09, 3.78)	0.98 (0.12, 9.04)	0.57 (0.06, 5.78)	0.53 (0.05, 5.47)	0.71 (0.07, 7.27)	0.43 (0.11, 1.63)	Osimertinib_Chemotherapy

Che, Chemotherapy.

#### 3.4.6 Local inconsistency analysis results

Node-splitting analysis was used to confirm the consistency of any two intervention schemes in any closed loop. This study analyzed the closed loops in four outcome indicators. For the PFS indicator, the indirect effect of Gefitinib alone compared to chemotherapy alone was more significant in improving PFS than the direct effect (P=0.038). Additionally, there were inconsistent estimates in the comparisons of Gefitinib + Chemotherapy with chemotherapy alone (P=0.036) and Gefitinib alone (P=0.034). For the OS indicator, the indirect effect of Gefitinib alone compared to chemotherapy alone significantly improved PFS (P=0.049). The indirect estimate of Gefitinib + Chemotherapy compared to Gefitinib alone was slightly higher than the direct comparison (P=0.464). In other closed loops and remaining outcome indicators, the P-values for the consistency between direct and indirect estimates were all greater than 0.05.

#### 3.4.7 Subgroup analysis

**Table 4** presents the results of subgroup network meta-analyses stratified by age (>65/≤65 years), EGFR mutation type (exon 19 deletion/21 L858R), and gender (male/female). The findings indicate that Osimertinib combined with chemotherapy demonstrates the most significant improvement in PFS among patients aged ≤65 years, those with exon 19 deletions, and females. In contrast, Gefitinib combined with chemotherapy emerges as a more effective treatment option for patients aged >65 years, those with the 21 L858R mutation, and males. The subgroup analysis network plots and ranking diagrams are provided in **Supplementary Figures 1** and **2**.

**Table 4:** Subgroup analysis results.

Subgroup	Intervention rank probability (%)		
	Rank 1^st^	Rank 2^nd^	Rank 3^rd^
Age				
≤65	Osimertinib+chemotherapy sucra: 78.9%	Osimertinibsucra: 67.0%	Gefitinib+chemotherapy sucra: 61.9%
>65	Gefitinib+chemotherapy sucra: 80.0%	Afatinib sucra: 73.7%	Icotinib+chemotherapy sucra: 60.0%
EGER mutation				
Del19	Osimertinib+chemotherapy sucra: 80.3%	Gefitinib+chemotherapy sucra: 75.1%	Afatinib sucra: 66.0%
21 L858R	Gefitinib+chemotherapy sucra: 85.8%	Osimertinib+chemotherapy sucra: 70.0%	Afatinib sucra: 60.0%
Gender				
Men	Gefitinib+chemotherapy sucra: 84.7%	Osimertinib+chemotherapy sucra: 67.8%	Icotinib+chemotherapy sucra: 60.0%
Women	Osimertinib+chemotherapy sucra: 76.8%	Gefitinib+chemotherapy sucra: 76.1%	Osimertinib sucra: 62.3%

## 4 Discussion

To our knowledge, this is the first NMA comparing the efficacy and safety of various first-line treatment options for advanced or metastatic EGFR mutation-positive NSCLC. The NMA analyzed the latest data from 30 eligible randomized controlled trials. Our findings indicate that Osimertinib + Chemotherapy is the most effective measure for improving PFS, Lazertinib is the best measure for improving OS, and Afatinib is the most prominent for enhancing ORR. Despite Afatinib's superior safety profile, the incidence of grade 3 or higher adverse events was not significantly different among all treatment regimens.

When considering PFS improvement, the top three treatments were Osimertinib + Chemotherapy, Osimertinib, and Osimertinib + Bevacizumab. Overall, Osimertinib, a third-generation EGFR-TKI, demonstrated outstanding performance. A previous network meta-analysis that included 17 trials indicated that Osimertinib-containing regimens were most likely to rank first in terms of PFS, regardless of the presence of brain metastases, which was consistent with our results (53). Additionally, a previous clinical trial found that the PFS of Osimertinib combined with Bevacizumab was shorter than that of Osimertinib alone in NSCLC patients with the EGFR T790M mutation (54), and we found the same result in an untreated population. Holleman et al. conducted a network meta-analysis that included data from 3539 EGFR mutation-positive NSCLC patients, comparing five EGFR-TKIs, including Afatinib, Dacomitinib, Erlotinib, Gefitinib, and Osimertinib in first-line treatment (55). The cumulative probability ranking indicated that Osimertinib had potentially better efficacy in terms of PFS compared to all other TKIs. Furthermore, the PFS improvement effect of Osimertinib combined with chemotherapy was the best, consistent with the effects observed by Hosomi et al. and Noronha et al. in their comparisons of first-generation EGFR-TKIs combined with or without chemotherapy in patients with advanced EGFR mutation-positive NSCLC (56, 57). Although the exact mechanism of the benefit of Osimertinib combined with chemotherapy remains unclear, as pointed out by the FLAURA-2 researchers, chemotherapy may complement Osimertinib's targeted action through its non-selective anti-tumor actions and overcome tumor heterogeneity (48).

Subgroup analyses of PFS under different population characteristics were conducted using network meta-analysis. Overall results indicate that Osimertinib combined with chemotherapy and Gefitinib combined with chemotherapy demonstrate optimal efficacy across subgroups stratified by age, EGFR mutation type, and gender. Among patients aged ≤65 years, those with exon 19 deletion mutations, or females, Osimertinib plus chemotherapy exhibited the most significant improvement in PFS. Previous real-world studies have identified factors associated with the efficacy of osimertinib, such as EGFR exon 19 deletions and female gender, which significantly enhance PFS outcomes with osimertinib treatment (58, 59). Additionally, a global Phase III trial on osimertinib in treatment-naïve EGFR-mutant NSCLC patients reported PFS of 21.4 months and 14.4 months for exon 19 deletion and L858R mutation patients, respectively, indicating a trend toward superior PFS in the exon 19 deletion group compared to the L858R group (12). In elderly patients, Gefitinib combined with chemotherapy was found to be the most effective treatment option, followed by Osimertinib combined with chemotherapy. In contrast, in younger patients, third-generation EGFR-TKIs (e.g., Osimertinib) combined with chemotherapy showed superior PFS improvement. Previous studies have revealed that elderly patients are more prone to lower BMI, which may compromise the efficacy of osimertinib due to potential cachexia—a condition characterized by significant weight loss primarily due to skeletal muscle and fat mass depletion (60, 61) (Furthermore, physiological decline in elderly patients may lead to reduced tolerance to potent drugs, making the lower-toxicity Gefitinib plus chemotherapy regimen a more suitable choice.

Regarding the improvement in OS, the top three treatment options were Lazertinib, Gefitinib, and Osimertinib + Chemotherapy. Lazertinib is a potent, orally available, blood-brain barrier-penetrable, irreversible third-generation EGFR-TKI. In our results, only Lazertinib showed a statistically significant improvement in OS compared to chemotherapy. Jeon et al. evaluated Lazertinib's efficacy against Osimertinib using external controls, finding that Lazertinib did not reach the median OS, whereas the Osimertinib group had a median OS of 29.8 months, suggesting Lazertinib's potential for superior survival benefits (62). Furthermore, Lee et al. reviewed and compared the efficacy outcomes of Lazertinib versus platinum-based chemotherapy in patients with EGFR mutation-positive NSCLC (63). Their results indicated that Lazertinib had excellent OS (32.23 months vs. 18.73 months; adjusted HR: 0.45, 95% CI: 0.29-0.69). In summary, Lazertinib may shows promise for OS improvement as a first-line treatment.

For ORR, Afatinib appears to be the best treatment option. Afatinib is a second-generation targeted drug that can covalently bind with the epidermal growth factor and other receptors, and it is irreversible. It effectively reduces medication time and prolongs the replacement probability of reversible non-covalent binding sites, thus inhibiting tyrosine kinase activity and the proliferation and metastasis of tumors. A review evaluating clinical outcomes in NSCLC patients with uncommon EGFR mutations found an ORR of 60.6% for the Afatinib group, slightly higher than 50.3% for the Osimertinib group, although the difference was not statistically significant (64). Another systematic review focusing on uncommon EGFR mutations included 38 studies with a total of 1836 patients, supporting the use of Afatinib for G719X, S768I, E709X, and L747X mutations, as well as compound rare mutations (65). Additionally, in a network meta-analysis conducted by Holleman et al., Afatinib and Osimertinib performed the best in ORR compared to other drugs (Dacomitinib, Erlotinib, Gefitinib), with a probability of 46% for both drugs (55). However, previous clinical trials have shown that approximately 60% of patients with acquired resistance to EGFR-TKIs (Erlotinib, Gefitinib, and Afatinib) develop a new mutation (T790M) in the drug target, which has been shown to alter drug binding and enzymatic activity of the mutant EGF receptor (66).

Regarding safety outcomes, all evaluated treatment options showed no statistically significant differences in the incidence of grade 3 or higher adverse events compared to chemotherapy or other regimens. Notably, Afatinib was identified as the most optimal treatment option in the cumulative probability ranking for safety, followed by Osimertinib. Previous real-world studies indicate that Afatinib is as effective and well-tolerated in routine clinical practice as in clinical trials, performing well in patients with certain uncommon EGFR mutations, brain metastases, and elderly patients (67, 68). Concerning Osimertinib, a clinical trial demonstrated that the incidence of grade 3 or higher adverse events in the Osimertinib group was similar to that in the control EGFR-TKI group (Gefitinib or Erlotinib), despite a longer exposure time in the Osimertinib group (13). Zhao et al. conducted a network meta-analysis that showed Icotinib had the highest probability (80%) of causing grade 3 or higher adverse events, followed by Osimertinib, although there was no statistically significant difference between Icotinib and Osimertinib (RR=2.92; 95% CI: 0.21-40.49). A meta-analysis of Osimertinib for advanced NSCLC with EGFR mutations revealed that the most common adverse events were diarrhea and rash, with combined incidences of 44% and 42%, respectively, followed by dry skin (29%). Moreover, most patients tolerated Osimertinib well (69).

### 4.1 Limitations

However, our study has several limitations. First, some trials in this study did not report or reach OS, potentially leading to a risk of bias in the results. Second, our NMA did not differentiate between specific forms of chemotherapy, and there may be efficacy differences between different chemotherapy regimens. Third, due to the limited number of current studies and the lack of OS subgroup analysis results from some studies, the data volume for subgroup analysis would be significantly reduced if considered, so we did not further explore subgroups to avoid impacting the power of the tests. Finally, since most trials were open-label, the lack of blinding might affect the credibility of the results.

## 5 Conclusion

In summary, our study indicates that Osimertinib is the preferred treatment option for untreated advanced or metastatic non-squamous NSCLC with EGFR mutations, considering both survival and safety. In particular, the combination of Osimertinib and chemotherapy demonstrates excellent survival benefits, providing crucial reference points for clinical decision-making for these patients. Nevertheless, our conclusions require further validation through more high-quality clinical studies with double-blind, multicenter, large-sample, and longer follow-up periods.

## Data Availability

The original contributions presented in the study are included in the article/**Supplementary Material**. Further inquiries can be directed to the corresponding author/s.

## Supplementary material

Supplementary Figure 1 Subgroup analysis Network map.

Supplementary Figure 2 Result of probability ranking for optimal PFS among different intervention measures in the subgroups. A, age; B, EGER mutation; C, gender.

Supplementary Text 1 Search strategy.
