# Supplementary figures and images for: First-line treatment for advanced or metastatic EGFR mutation-positive non-squamous non-small cell lung cancer: a network meta-analysis
Source: Front Oncol. 2025 Jan 15;14:1498518. doi: 10.3389/fonc.2024.1498518 (PMC11774708; doi:10.3389/fonc.2024.1498518)

A

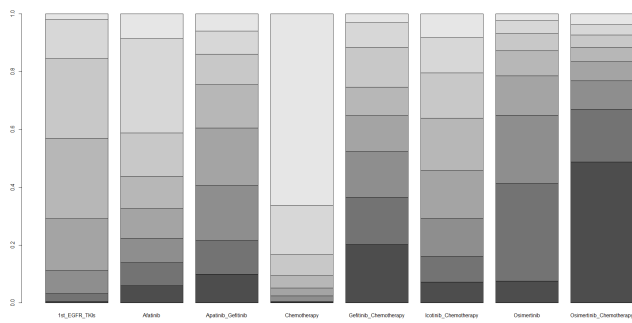

Age (<65)

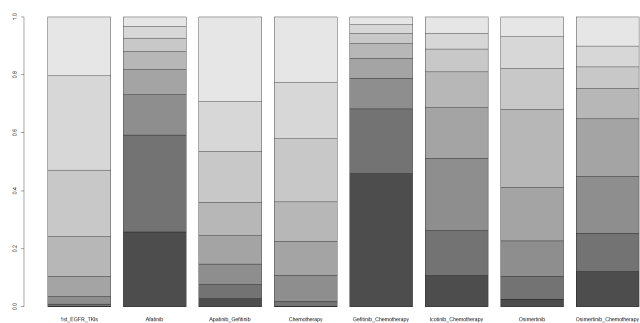

Age (>=65)

B

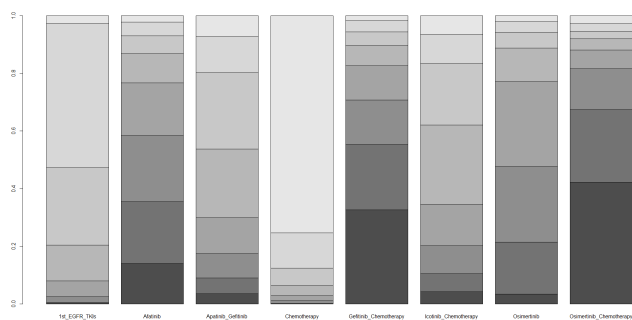

EGER mutation (Del19)

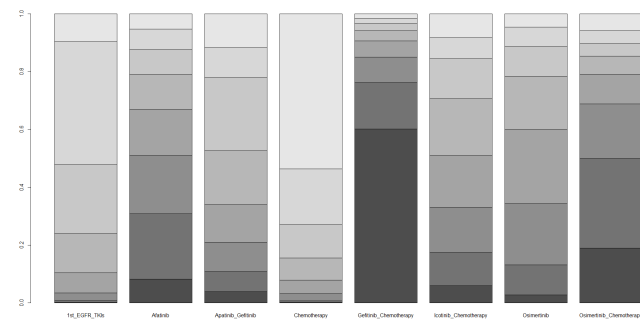

EGER mutation (21 L858R)

C

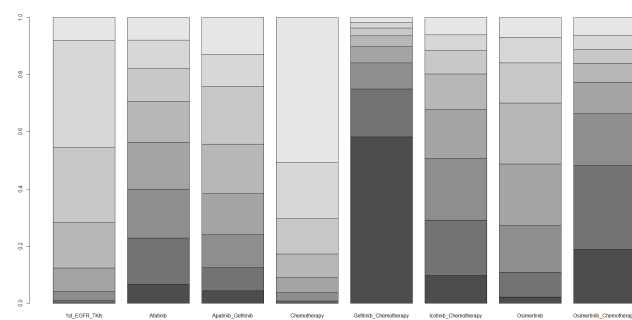

sex (men)

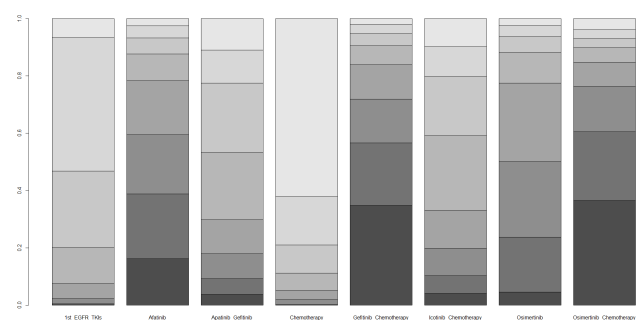

sex (women)

Supplement: Supplementary Figure 1 — Subgroup analysis Network map. [file DataSheet1.pdf]

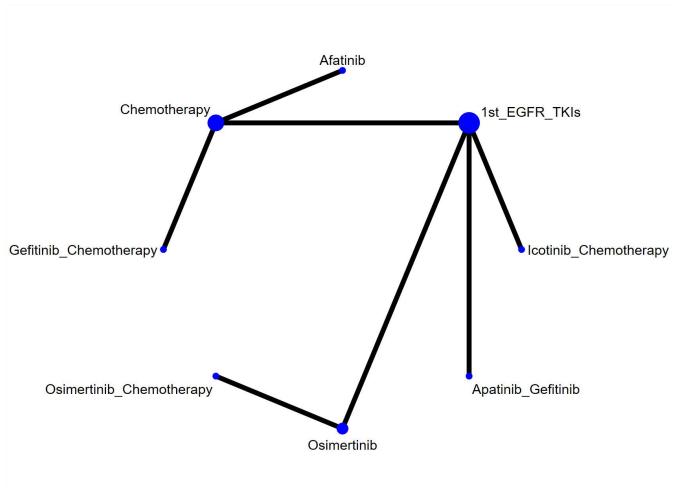

A: Age (<65 or ≥65)

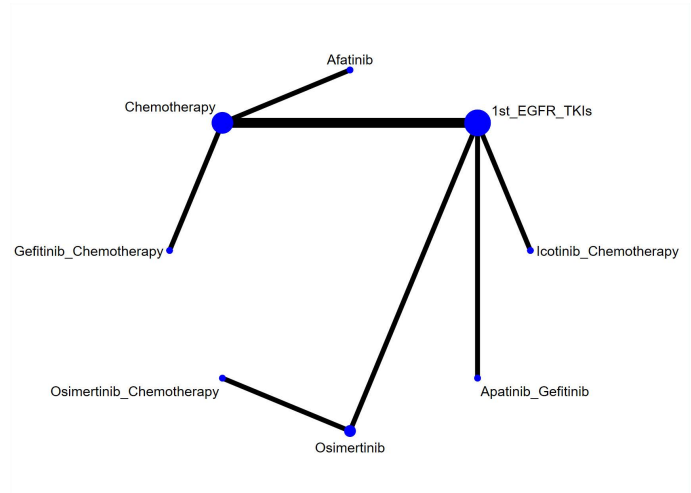

B: EGER mutation (Del19 or 21 L858R)

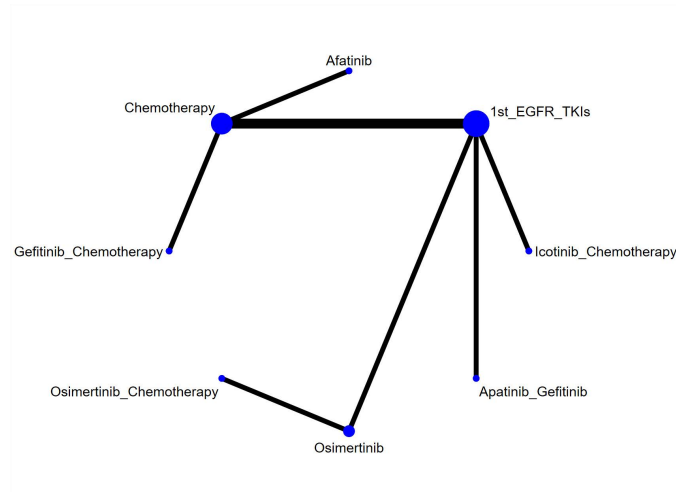

C: sex (men or women)

Supplement: Supplementary Figure 2 — Result of probability ranking for optimal PFS among different intervention measures in the subgroups. A, age; B, EGER mutation; C, gender. [file DataSheet2.pdf]
