# Supplementary material for: First-line treatment for advanced or metastatic EGFR mutation-positive non-squamous non-small cell lung cancer: a network meta-analysis
Source: Front Oncol. 2025 Jan 15;14:1498518. doi: 10.3389/fonc.2024.1498518 (PMC11774708; doi:10.3389/fonc.2024.1498518)
Supplement: Supplementary Text 1 — Search strategy. [file DataSheet3.docx]

# Supplementary Text 1 Literature search strategy

**1.Pubmed**

| Search number | Query |
| --- | --- |
| #1 | "Carcinoma, Non-Small-Cell Lung"[Mesh] |
| #2 | (NSCLC[Title/Abstract]) OR ("Carcinoma, Non-Small-Cell Lung"[Mesh]) |
| #3 | "non-small cell "[Title/Abstract] OR "non small-cell "[Title/Abstract] OR "non small cell "[Title/Abstract] OR "nonsmall cell "[Title/Abstract] |
| #4 | "lung"[Title/Abstract] |
| #5 | ("non-small cell "[Title/Abstract] OR "non small-cell "[Title/Abstract] OR "non small cell "[Title/Abstract] OR "nonsmall cell "[Title/Abstract]) AND ("lung"[Title/Abstract]) |
| #6 | (("non-small cell "[Title/Abstract] OR "non small-cell "[Title/Abstract] OR "non small cell "[Title/Abstract] OR "nonsmall cell "[Title/Abstract]) AND ("lung"[Title/Abstract])) OR ((NSCLC[Title/Abstract]) OR ("Carcinoma, Non-Small-Cell Lung"[Mesh])) |
| #7 | "epidermal growth factor receptor"[Title/Abstract] OR "EGFR"[Title/Abstract] OR "tyrosine kinase inhibit"[Title/Abstract] OR "TKI"[Title/Abstract] OR "erlotinib"[Title/Abstract] OR "tarceva"[Title/Abstract] OR "gefitinib"[Title/Abstract] OR "iressa"[Title/Abstract] OR "afatinib"[Title/Abstract] OR "gilotrif"[Title/Abstract] OR "osimertinib"[Title/Abstract] OR "Tagrisso"[Title/Abstract] OR "mereletinib"[Title/Abstract] OR "dacomitinib"[Title/Abstract] OR "icotinib"[Title/Abstract] |
| #8 | "advance*"[Title/Abstract] OR "Metastatic*"[Title/Abstract] OR "III"[Title/Abstract] OR "IV"[Title/Abstract] |
| #9 | "first-line"[Title/Abstract] OR "first line"[Title/Abstract] OR "untreated"[Title/Abstract] OR "treatment-naïve"[Title/Abstract] OR "treatment naïve"[Title/Abstract] OR "chemo naïve"[Title/Abstract] OR "front line"[Title/Abstract] |
| #10 | "ErbB Receptors"[Mesh] |
| #11 | ("epidermal growth factor receptor"[Title/Abstract] OR "EGFR"[Title/Abstract] OR "tyrosine kinase inhibit"[Title/Abstract] OR "TKI"[Title/Abstract] OR "erlotinib"[Title/Abstract] OR "tarceva"[Title/Abstract] OR "gefitinib"[Title/Abstract] OR "iressa"[Title/Abstract] OR "afatinib"[Title/Abstract] OR "gilotrif"[Title/Abstract] OR "osimertinib"[Title/Abstract] OR "Tagrisso"[Title/Abstract] OR "mereletinib"[Title/Abstract] OR "dacomitinib"[Title/Abstract] OR "icotinib"[Title/Abstract]) OR ("ErbB Receptors"[Mesh]) |
| #12 | ((((("non-small cell "[Title/Abstract] OR "non small-cell "[Title/Abstract] OR "non small cell "[Title/Abstract] OR "nonsmall cell "[Title/Abstract]) AND ("lung"[Title/Abstract])) OR ((NSCLC[Title/Abstract]) OR ("Carcinoma, Non-Small-Cell Lung"[Mesh]))) AND (("epidermal growth factor receptor"[Title/Abstract] OR "EGFR"[Title/Abstract] OR "tyrosine kinase inhibit"[Title/Abstract] OR "TKI"[Title/Abstract] OR "erlotinib"[Title/Abstract] OR "tarceva"[Title/Abstract] OR "gefitinib"[Title/Abstract] OR "iressa"[Title/Abstract] OR "afatinib"[Title/Abstract] OR "gilotrif"[Title/Abstract] OR "osimertinib"[Title/Abstract] OR "Tagrisso"[Title/Abstract] OR "mereletinib"[Title/Abstract] OR "dacomitinib"[Title/Abstract] OR "icotinib"[Title/Abstract]) OR ("ErbB Receptors"[Mesh]))) AND ("advance*"[Title/Abstract] OR "Metastatic*"[Title/Abstract] OR "III"[Title/Abstract] OR "IV"[Title/Abstract])) AND ("first-line"[Title/Abstract] OR "first line"[Title/Abstract] OR "untreated"[Title/Abstract] OR "treatment-naïve"[Title/Abstract] OR "treatment naïve"[Title/Abstract] OR "chemo naïve"[Title/Abstract] OR "front line"[Title/Abstract]) |

**2.Cochrane**

| Search number | Query |
| --- | --- |
| #1 | MeSH descriptor: [Carcinoma, Non-Small-Cell Lung] explode all trees |
| #2 | (NSCLC):ti,ab,kw |
| #3 | (non-small cell ):ti,ab,kw OR (non small-cell ):ti,ab,kw OR (non small cell ):ti,ab,kw OR (nonsmall cell ):ti,ab,kw |
| #4 | (lung):ti,ab,kw |
| #5 | #1 or #2 |
| #6 | #3 and #4 |
| #7 | #5 or #6 |
| #8 | (epidermal growth factor receptor):ti,ab,kw OR (EGFR):ti,ab,kw OR (tyrosine kinase inhibit):ti,ab,kw OR (TKI):ti,ab,kw OR (erlotinib):ti,ab,kw OR (tarceva):ti,ab,kw OR (gefitinib):ti,ab,kw OR (iressa):ti,ab,kw OR (afatinib):ti,ab,kw OR (gilotrif):ti,ab,kw OR (osimertinib):ti,ab,kw OR (Tagrisso):ti,ab,kw OR (mereletinib):ti,ab,kw OR (dacomitinib):ti,ab,kw OR (icotinib):ti,ab,kw |
| #9 | MeSH descriptor: [ErbB Receptors] explode all trees |
| #10 | #8 or #9 |
| #11 | (advance*):ti,ab,kw OR (Metastatic*):ti,ab,kw OR (III):ti,ab,kw OR (IV):ti,ab,kw |
| #12 | (first-line):ti,ab,kw OR (first line):ti,ab,kw OR (untreated):ti,ab,kw OR (treatment-naïve):ti,ab,kw OR (treatment naïve):ti,ab,kw OR (chemo naïve):ti,ab,kw OR (front line):ti,ab,kw |
| #13 | #7 and #10 and #11 and #12 |

**3.Embase**

| Search number | Query |
| --- | --- |
| #1 | 'non small cell lung cancer'/exp |
| #2 | nsclc:ab,ti |
| #3 | #1 OR #2 |
| #4 | 'non-small cell':ab,ti OR 'non small-cell':ab,ti OR 'non small cell':ab,ti OR 'nonsmall cell':ab,ti |
| #5 | 'lung':ab,ti |
| #6 | #4 AND #5 |
| #7 | #3 OR #6 |
| #8 | 'epidermal growth factor receptor'/exp |
| #9 | 'epidermal growth factor receptor':ab,ti OR 'egfr':ab,ti OR 'tyrosine kinase inhibit':ab,ti OR 'tki':ab,ti OR 'erlotinib':ab,ti OR 'tarceva':ab,ti OR 'gefitinib':ab,ti OR 'iressa':ab,ti OR 'afatinib':ab,ti OR 'gilotrif':ab,ti OR 'osimertinib':ab,ti OR 'tagrisso':ab,ti OR 'mereletinib':ab,ti OR 'dacomitinib':ab,ti OR 'icotinib':ab,ti |
| #10 | #8 OR #9 |
| #11 | 'advance*':ab,ti OR 'metastatic*':ab,ti OR 'iii':ab,ti OR 'iv':ab,ti |
| #12 | 'first-line':ab,ti OR 'first line':ab,ti OR 'untreated':ab,ti OR 'treatment- naïve':ab,ti OR 'treatment naïve':ab,ti OR 'chemo naïve':ab,ti OR 'front line':ab,ti |
| #13 | #7 AND #10 AND #11 AND #12 |

**4.Web of science**

| Search number | Query |
| --- | --- |
| #1 | NSCLC (Topic) |
| #2 | TS=(non-small cell ) OR TS=(non small-cell ) OR TS=(non small cell ) OR TS=(nonsmall cell ) |
| #3 | TS=(lung) |
| #4 | #2 AND #3 |
| #5 | #4 OR #1 |
| #6 | TS=(epidermal growth factor receptor) OR TS=(EGFR) OR TS=(tyrosine kinase inhibit) OR TS=(TKI) OR TS=(erlotinib) OR TS=(tarceva) OR TS=(gefitinib) OR TS=(iressa) OR TS=(afatinib) OR TS=(gilotrif) OR TS=(osimertinib) OR TS=(Tagrisso) OR TS=(mereletinib) OR TS=(dacomitinib) OR TS=(icotinib) |
| #7 | TS=(advance*) OR TS=(Metastatic*) OR TS=(III) OR TS=(IV) |
| #8 | TS=(first-line) OR TS=(first line) OR TS=(untreated) OR TS=(treatment-naïve) OR TS=(treatment naïve) OR TS=(chemo naïve) OR TS=(front line) |
| #9 | #5 AND #6 AND #7 AND #8 |
