# Supplementary material for: First-line treatment for advanced or metastatic EGFR mutation-positive non-squamous non-small cell lung cancer: a network meta-analysis
Source: Front Oncol. 2025 Jan 15;14:1498518. doi: 10.3389/fonc.2024.1498518 (PMC11774708; doi:10.3389/fonc.2024.1498518)
Supplement: Supplementary file 4 [file Table1.docx]

**Supplements Table 1:** The OS league table. Che, Chemotherapy.

|  | Afatinib | Afatinib_Cetuximab | Apatinib_Gefitinib | Befotertinib | Cetuximab_Che | Che | Erlotinib | Erlotinib_Bevacizumab | Erlotinib_Che | Gefitinib | Gefitinib_Che | Gefitinib_Olaparib | Icotinib | Icotinib_Che | Lazertinib | Osimertinib | Osimertinib_Che |
| --- | --- | --- | --- | --- | --- | --- | --- | --- | --- | --- | --- | --- | --- | --- | --- | --- | --- |
| Afatinib | Afatinib | 1.45(0.55,3.86) | 0.92(0.48,1.77) | 1.27(0.61,2.68) | 1.01(0.68,1.49) | 1.11(0.83,1.47) | 1.42(0.82,2.51) | 1.69(0.84,3.52) | 1.1(0.44,2.63) | 0.84(0.56,1.25) | 0.83(0.48,1.47) | 1.02(0.53,1.94) | 1.09(0.64,1.88) | 0.89(0.42,1.86) | 0.62(0.33,1.16) | 0.9(0.44,1.86) | 0.8(0.35,1.88) |
| Afatinib_Cetuximab | 0.69(0.26,1.83) | Afatinib_Cetuximab | 0.63(0.19,2.07) | 0.88(0.25,3.06) | 0.7(0.24,1.99) | 0.76(0.27,2.12) | 0.98(0.31,3.03) | 1.17(0.35,3.94) | 0.76(0.2,2.69) | 0.58(0.2,1.67) | 0.57(0.19,1.86) | 0.71(0.21,2.25) | 0.75(0.24,2.31) | 0.61(0.17,2.1) | 0.43(0.13,1.36) | 0.62(0.18,2.08) | 0.55(0.15,2.01) |
| Apatinib_Gefitinib | 1.09(0.57,2.1) | 1.58(0.48,5.19) | Apatinib_Gefitinib | 1.38(0.57,3.42) | 1.1(0.58,2.09) | 1.2(0.67,2.17) | 1.54(0.72,3.3) | 1.84(0.77,4.47) | 1.19(0.42,3.29) | 0.91(0.54,1.52) | 0.9(0.45,1.85) | 1.1(0.53,2.29) | 1.18(0.57,2.51) | 0.96(0.4,2.36) | 0.68(0.33,1.35) | 0.97(0.41,2.35) | 0.87(0.33,2.37) |
| Befotertinib | 0.78(0.37,1.63) | 1.14(0.33,4) | 0.72(0.29,1.77) | Befotertinib | 0.79(0.38,1.64) | 0.87(0.44,1.71) | 1.11(0.48,2.58) | 1.32(0.52,3.45) | 0.86(0.29,2.53) | 0.66(0.31,1.36) | 0.65(0.28,1.5) | 0.8(0.33,1.94) | 0.86(0.52,1.39) | 0.7(0.35,1.39) | 0.49(0.2,1.18) | 0.7(0.27,1.82) | 0.63(0.22,1.79) |
| Cetuximab_Che | 0.99(0.67,1.47) | 1.43(0.5,4.13) | 0.91(0.48,1.74) | 1.26(0.61,2.62) | Cetuximab_Che | 1.09(0.84,1.43) | 1.41(0.82,2.47) | 1.68(0.83,3.46) | 1.08(0.44,2.57) | 0.83(0.56,1.21) | 0.82(0.48,1.43) | 1.01(0.53,1.9) | 1.08(0.64,1.84) | 0.88(0.42,1.83) | 0.62(0.33,1.15) | 0.88(0.44,1.83) | 0.79(0.35,1.85) |
| Che | 0.9(0.68,1.21) | 1.31(0.47,3.64) | 0.83(0.46,1.5) | 1.16(0.58,2.28) | 0.92(0.7,1.19) | Che | 1.29(0.8,2.12) | 1.54(0.8,3) | 0.99(0.42,2.28) | 0.76(0.57,1) | 0.75(0.47,1.22) | 0.92(0.51,1.65) | 0.99(0.62,1.57) | 0.8(0.41,1.6) | **0.56(0.32,0.99)** | 0.81(0.42,1.58) | 0.73(0.33,1.63) |
| Erlotinib | 0.7(0.4,1.23) | 1.02(0.33,3.19) | 0.65(0.3,1.39) | 0.9(0.39,2.09) | 0.71(0.4,1.23) | 0.78(0.47,1.25) | Erlotinib | 1.19(0.77,1.85) | 0.77(0.39,1.51) | 0.59(0.33,1.02) | 0.58(0.3,1.17) | 0.72(0.33,1.51) | 0.77(0.39,1.5) | 0.62(0.27,1.43) | **0.44(0.21,0.9)** | **0.63(0.4,0.98)** | 0.56(0.3,1.06) |
| Erlotinib_Bevacizumab | 0.59(0.28,1.19) | 0.85(0.25,2.88) | 0.54(0.22,1.31) | 0.75(0.29,1.92) | 0.6(0.29,1.2) | 0.65(0.33,1.25) | 0.84(0.54,1.3) | Erlotinib_Bevacizumab | 0.65(0.29,1.46) | 0.5(0.24,1) | 0.49(0.22,1.12) | 0.6(0.24,1.43) | 0.65(0.28,1.43) | 0.52(0.2,1.32) | **0.37(0.15,0.85)** | **0.53(0.28,0.98)** | 0.47(0.22,1.01) |
| Erlotinib_Che | 0.91(0.38,2.25) | 1.32(0.37,4.95) | 0.84(0.3,2.38) | 1.16(0.39,3.5) | 0.92(0.39,2.25) | 1.01(0.44,2.36) | 1.3(0.66,2.57) | 1.54(0.69,3.46) | Erlotinib_Che | 0.76(0.32,1.88) | 0.76(0.29,2.01) | 0.92(0.34,2.59) | 0.99(0.38,2.62) | 0.81(0.27,2.39) | 0.57(0.21,1.58) | 0.82(0.36,1.84) | 0.73(0.29,1.84) |
| Gefitinib | 1.19(0.8,1.8) | 1.73(0.6,5.03) | 1.1(0.66,1.85) | 1.52(0.73,3.2) | 1.2(0.82,1.78) | 1.32(1,1.76) | 1.69(0.98,2.99) | 2.02(1,4.18) | 1.31(0.53,3.12) | Gefitinib | 0.99(0.62,1.59) | 1.21(0.73,2.03) | 1.3(0.76,2.26) | 1.05(0.51,2.24) | 0.74(0.46,1.2) | 1.06(0.52,2.2) | 0.96(0.42,2.25) |
| Gefitinib_Che | 1.21(0.68,2.08) | 1.76(0.54,5.39) | 1.11(0.54,2.21) | 1.54(0.67,3.51) | 1.22(0.7,2.08) | 1.34(0.82,2.15) | 1.72(0.86,3.36) | 2.04(0.9,4.58) | 1.32(0.5,3.44) | 1.01(0.63,1.61) | Gefitinib_Che | 1.22(0.61,2.42) | 1.31(0.67,2.54) | 1.07(0.46,2.48) | 0.75(0.38,1.48) | 1.08(0.48,2.43) | 0.97(0.38,2.44) |
| Gefitinib_Olaparib | 0.98(0.52,1.89) | 1.42(0.45,4.66) | 0.91(0.44,1.88) | 1.25(0.52,3.05) | 0.99(0.53,1.88) | 1.08(0.61,1.96) | 1.39(0.66,3.02) | 1.66(0.7,4.09) | 1.09(0.39,2.96) | 0.82(0.49,1.38) | 0.82(0.41,1.63) | Gefitinib_Olaparib | 1.07(0.51,2.27) | 0.87(0.36,2.17) | 0.61(0.3,1.24) | 0.88(0.37,2.14) | 0.79(0.3,2.13) |
| Icotinib | 0.92(0.53,1.57) | 1.33(0.43,4.15) | 0.85(0.4,1.77) | 1.17(0.72,1.91) | 0.92(0.54,1.57) | 1.01(0.64,1.61) | 1.3(0.67,2.58) | 1.55(0.7,3.53) | 1.01(0.38,2.63) | 0.77(0.44,1.31) | 0.76(0.39,1.49) | 0.93(0.44,1.97) | Icotinib | 0.81(0.49,1.33) | 0.57(0.27,1.18) | 0.82(0.37,1.86) | 0.74(0.29,1.86) |
| Icotinib_Che | 1.12(0.54,2.36) | 1.64(0.48,5.78) | 1.04(0.42,2.52) | 1.44(0.72,2.89) | 1.14(0.55,2.35) | 1.24(0.63,2.46) | 1.6(0.7,3.77) | 1.91(0.76,5) | 1.23(0.42,3.71) | 0.95(0.45,1.96) | 0.94(0.4,2.15) | 1.15(0.46,2.81) | 1.23(0.75,2.03) | Icotinib_Che | 0.7(0.28,1.69) | 1.01(0.4,2.61) | 0.91(0.32,2.58) |
| Lazertinib | 1.61(0.86,3.03) | 2.34(0.73,7.68) | 1.48(0.74,3.01) | 2.05(0.85,5.05) | 1.62(0.87,3.01) | **1.78(1.01,3.11)** | **2.29(1.11,4.84)** | **2.71(1.17,6.61)** | 1.76(0.63,4.85) | 1.35(0.83,2.18) | 1.34(0.67,2.65) | 1.64(0.81,3.3) | 1.76(0.85,3.67) | 1.43(0.59,3.51) | Lazertinib | 1.43(0.61,3.45) | 1.29(0.5,3.47) |
| Osimertinib | 1.12(0.54,2.29) | 1.62(0.48,5.45) | 1.03(0.43,2.46) | 1.42(0.55,3.71) | 1.13(0.55,2.28) | 1.24(0.63,2.39) | **1.59(1.02,2.47)** | **1.89(1.02,3.53)** | 1.23(0.54,2.76) | 0.94(0.45,1.91) | 0.93(0.41,2.1) | 1.14(0.47,2.69) | 1.22(0.54,2.71) | 0.99(0.38,2.52) | 0.7(0.29,1.63) | Osimertinib | 0.9(0.58,1.4) |
| Osimertinib_Che | 1.25(0.53,2.88) | 1.81(0.5,6.65) | 1.14(0.42,3.04) | 1.59(0.56,4.57) | 1.26(0.54,2.89) | 1.38(0.61,3.03) | 1.78(0.95,3.3) | 2.11(0.99,4.55) | 1.37(0.54,3.48) | 1.05(0.44,2.4) | 1.03(0.41,2.63) | 1.27(0.47,3.34) | 1.36(0.54,3.4) | 1.1(0.39,3.13) | 0.78(0.29,1.99) | 1.11(0.72,1.71) | Osimertinib_Che |
