# Supplementary material for: First-line treatment for advanced or metastatic EGFR mutation-positive non-squamous non-small cell lung cancer: a network meta-analysis
Source: Front Oncol. 2025 Jan 15;14:1498518. doi: 10.3389/fonc.2024.1498518 (PMC11774708; doi:10.3389/fonc.2024.1498518)
Supplement: Supplementary file 5 [file Table2.docx]

**Supplement Table 2:** The ORR league table. Che, Chemotherapy.

|  | Afatinib | Afatinib_Cetuximab | Apatinib_Gefitinib | Befotertinib | Cetuximab_Che | Che | Gefitinib | Gefitinib_Che | Gefitinib_Olaparib | Icotinib | Icotinib_Che | Lazertinib | Naquotinib | Osimertinib | Osimertinib_Che |
| --- | --- | --- | --- | --- | --- | --- | --- | --- | --- | --- | --- | --- | --- | --- | --- |
| Afatinib | Afatinib | 1.01(0.63,1.61) | 0.6(0.33,1.18) | 0.53(0.24,1.15) | **0.38(0.21,0.71)** | **0.37(0.25,0.53)** | **0.58(0.37,0.94)** | 0.68(0.42,1.16) | 0.61(0.32,1.2) | 0.54(0.28,1.05) | 0.66(0.3,1.47) | 0.58(0.31,1.12) | **0.4(0.21,0.78)** | 0.59(0.32,1.15) | 0.65(0.32,1.46) |
| Afatinib_Cetuximab | 0.99(0.62,1.58) | Afatinib_Cetuximab | 0.6(0.28,1.35) | 0.52(0.21,1.29) | **0.38(0.18,0.82)** | **0.36(0.2,0.66)** | 0.57(0.3,1.13) | 0.67(0.34,1.38) | 0.6(0.28,1.38) | 0.54(0.24,1.2) | 0.66(0.26,1.65) | 0.57(0.27,1.3) | **0.39(0.18,0.91)** | 0.58(0.28,1.33) | 0.65(0.27,1.65) |
| Apatinib_Gefitinib | 1.66(0.85,3.04) | 1.68(0.74,3.58) | Apatinib_Gefitinib | 0.87(0.36,2) | 0.64(0.3,1.25) | **0.61(0.35,0.98)** | 0.96(0.61,1.49) | 1.13(0.64,1.97) | 1(0.53,1.9) | 0.9(0.42,1.84) | 1.1(0.44,2.54) | 0.95(0.51,1.79) | 0.66(0.35,1.25) | 0.98(0.53,1.83) | 1.09(0.51,2.33) |
| Befotertinib | 1.9(0.87,4.19) | 1.92(0.78,4.79) | 1.14(0.5,2.82) | Befotertinib | 0.73(0.32,1.71) | 0.69(0.35,1.39) | 1.1(0.54,2.38) | 1.29(0.61,2.89) | 1.15(0.5,2.84) | 1.03(0.67,1.61) | 1.26(0.67,2.38) | 1.09(0.48,2.68) | 0.75(0.33,1.86) | 1.12(0.5,2.74) | 1.24(0.5,3.39) |
| Cetuximab_Che | **2.6(1.41,4.83)** | **2.63(1.22,5.69)** | 1.57(0.8,3.28) | 1.37(0.58,3.17) | Cetuximab_Che | 0.95(0.58,1.55) | 1.5(0.89,2.69) | 1.77(1,3.3) | 1.58(0.79,3.34) | 1.42(0.69,2.93) | 1.73(0.73,4.07) | 1.5(0.76,3.13) | 1.03(0.51,2.18) | 1.54(0.79,3.2) | 1.7(0.78,4.02) |
| Che | **2.73(1.88,3.98)** | **2.77(1.52,5.02)** | **1.65(1.02,2.85)** | 1.44(0.72,2.85) | 1.05(0.64,1.71) | Che | **1.58(1.24,2.12)** | **1.86(1.35,2.7)** | 1.66(1,2.89) | 1.49(0.87,2.56) | 1.81(0.89,3.66) | 1.57(0.97,2.72) | 1.08(0.65,1.91) | 1.61(1,2.79) | 1.79(0.95,3.62) |
| Gefitinib | **1.73(1.06,2.68)** | 1.75(0.88,3.28) | 1.05(0.67,1.63) | 0.91(0.42,1.85) | 0.67(0.37,1.13) | **0.63(0.47,0.8)** | Gefitinib | 1.18(0.83,1.68) | 1.05(0.66,1.66) | 0.94(0.5,1.68) | 1.15(0.53,2.38) | 1(0.65,1.55) | 0.69(0.43,1.09) | 1.03(0.66,1.59) | 1.14(0.62,2.11) |
| Gefitinib_Che | 1.47(0.86,2.4) | 1.48(0.73,2.9) | 0.89(0.51,1.57) | 0.77(0.35,1.63) | 0.56(0.3,1) | **0.54(0.37,0.74)** | 0.85(0.6,1.21) | Gefitinib_Che | 0.89(0.5,1.59) | 0.8(0.41,1.49) | 0.97(0.43,2.09) | 0.85(0.49,1.49) | 0.58(0.33,1.05) | 0.87(0.5,1.53) | 0.96(0.48,1.97) |
| Gefitinib_Olaparib | 1.65(0.84,3.09) | 1.67(0.73,3.6) | 1(0.53,1.9) | 0.87(0.35,2.01) | 0.63(0.3,1.27) | 0.6(0.35,1) | 0.95(0.6,1.51) | 1.12(0.63,2.01) | Gefitinib_Olaparib | 0.9(0.41,1.86) | 1.09(0.44,2.57) | 0.95(0.51,1.81) | 0.65(0.34,1.27) | 0.98(0.52,1.84) | 1.08(0.5,2.34) |
| Icotinib | 1.84(0.95,3.55) | 1.86(0.83,4.16) | 1.11(0.54,2.41) | 0.97(0.62,1.5) | 0.71(0.34,1.46) | 0.67(0.39,1.15) | 1.06(0.59,1.99) | 1.25(0.67,2.43) | 1.11(0.54,2.44) | Icotinib | 1.21(0.77,1.94) | 1.06(0.52,2.3) | 0.73(0.35,1.6) | 1.09(0.54,2.34) | 1.2(0.53,2.94) |
| Icotinib_Che | 1.51(0.68,3.36) | 1.53(0.61,3.85) | 0.91(0.39,2.26) | 0.8(0.42,1.5) | 0.58(0.25,1.37) | 0.55(0.27,1.12) | 0.87(0.42,1.9) | 1.03(0.48,2.31) | 0.91(0.39,2.28) | 0.82(0.52,1.3) | Icotinib_Che | 0.87(0.38,2.14) | 0.6(0.26,1.49) | 0.89(0.39,2.2) | 0.99(0.39,2.7) |
| Lazertinib | 1.74(0.9,3.19) | 1.76(0.77,3.74) | 1.05(0.56,1.95) | 0.92(0.37,2.08) | 0.67(0.32,1.31) | 0.64(0.37,1.03) | 1(0.64,1.55) | 1.18(0.67,2.06) | 1.05(0.55,1.98) | 0.95(0.43,1.92) | 1.15(0.47,2.66) | Lazertinib | 0.69(0.36,1.3) | 1.03(0.55,1.9) | 1.14(0.54,2.42) |
| Naquotinib | **2.53(1.28,4.72)** | **2.56(1.1,5.53)** | 1.52(0.8,2.88) | 1.33(0.54,3.06) | 0.97(0.46,1.94) | 0.92(0.52,1.53) | 1.46(0.92,2.32) | 1.72(0.95,3.07) | 1.53(0.79,2.95) | 1.37(0.62,2.85) | 1.68(0.67,3.9) | 1.46(0.77,2.76) | Naquotinib | 1.49(0.79,2.83) | 1.66(0.77,3.59) |
| Osimertinib | 1.69(0.87,3.08) | 1.71(0.75,3.61) | 1.02(0.55,1.89) | 0.89(0.36,2.02) | 0.65(0.31,1.27) | 0.62(0.36,1) | 0.97(0.63,1.51) | 1.15(0.65,2) | 1.02(0.54,1.93) | 0.92(0.43,1.87) | 1.12(0.45,2.58) | 0.97(0.53,1.81) | 0.67(0.35,1.27) | Osimertinib | 1.11(0.72,1.71) |
| Osimertinib_Che | 1.53(0.69,3.15) | 1.54(0.61,3.64) | 0.92(0.43,1.96) | 0.8(0.3,2) | 0.59(0.25,1.29) | 0.56(0.28,1.05) | 0.88(0.47,1.62) | 1.04(0.51,2.09) | 0.92(0.43,2) | 0.83(0.34,1.88) | 1.01(0.37,2.56) | 0.88(0.41,1.87) | 0.6(0.28,1.3) | 0.9(0.58,1.39) | Osimertinib_Che |
